# First aid self-efficacy: a scale adaptation and psychometric properties

**DOI:** 10.1186/s12889-025-22486-w

**Published:** 2025-04-01

**Authors:** Minna Sihvo, Ville Heilala, Tommi Kärkkäinen

**Affiliations:** 1 Faculty of Information Technology, University of Jyväskylä, PL 35, Jyväskylä, 40014 Finland; 2 Faculty of Humanities and Social Sciences, University of Jyväskylä, PL 35, Jyväskylä, 40014 Finland

**Keywords:** First aid, Helping behavior, Self-efficacy, Scale, Psychometric properties

## Abstract

**Background:**

Self-efficacy is a crucial predictor of effective performance in medical emergencies requiring first aid skills. Despite this, there is no standardized instrument for reliably measuring self-efficacy in first aid situations. The aim was to fill this gap by developing a novel first aid self-efficacy scale and validate it through a comprehensive assessment of its psychometric properties.

**Methods:**

A systematic assessment of the existing first aid self-efficacy scales was conducted. A psychometric analysis process involving 1152 participants was undertaken for the new scale. The analysis utilized factor analysis, non-parametric item response theory, and classical test theory, including validity assessment.

**Results:**

The developed first aid self-efficacy scale demonstrated excellent psychometric properties. The scale exhibited a robust internal structure, high reliability, and strong construct validity. It showed significant positive correlations with related constructs and effectively distinguished between different levels of first aid knowledge and training history.

**Conclusion:**

The first aid self-efficacy scale is a novel, reliable, and valid instrument for assessing self-efficacy in first aid contexts. It can be used to measure the impact of first aid training and interventions, thereby promoting more effective layperson responses in emergencies. The scale’s robust psychometric properties make it a valuable tool for both research and practical applications in emergency preparedness and first aid training.

**Supplementary Information:**

The online version contains supplementary material available at 10.1186/s12889-025-22486-w.

## Introduction

Accidents in traffic, workplaces, and homes, as well as during leisure time, constitute a significant public health issue. For instance, within the EU in 2019, the number of non-fatal accidents was 2.88 million and the number of fatal accidents 151,337, which constitutes 3.3 % of all deaths [1]. In particular, one of the major causes of death was road accidents, with the number of deaths being around 1.35 million in 2018 worldwide and almost 20,000 in the EU in 2021, respectively [2, 3]. On the other hand, in the United States, trauma is the leading cause of death for individuals younger than 45 years, and uncontrolled hemorrhage is a major cause of trauma mortality [4, 5]. In all these situations, the arrival time of an emergency has a decisive role in preventing deaths [6]. Before the arrival of the emergency medical care, the provision of first aid is up to the immediate responders who are first on the scene. Without immediate responders’ ability to help, the number and proportion of potentially preventable prehospital deaths remain high in different medical emergencies [3, 7].

Indeed, the first aid given by an immediate responder has been shown to save lives and to reduce disabilities in emergency situations [8–12]. The proper actions are necessary because it has been estimated that 40–60 % of the victims are left without the necessary help [13–15]. Therefore, many attempts have been made to prevent and reduce the consequences of different injuries and accidents by developing optimal emergency preparedness strategies and guidelines for laypeople [4, 16–21]. One widespread strategy is providing first aid guidelines to enable immediate responders to cope with all common emergencies, including accidents. According to the European Guidelines, the goals of first aid include preserving life, alleviating suffering, preventing further illness or injury, and promoting recovery [18].

First aid skills are often viewed as psychomotor skills such as Cardio Pulmonary Resuscitation (CPR) or knowledge retention capabilities [22, 23]. Emergency situations, however, can be demanding and frightening for laypeople. Many barriers, such as the fear of making mistakes and hurting victims or a lack of first aid training, can prevent laypeople from acting [16, 24–27]. Therefore, emotions are strongly involved in first aid situations. The core emotional factor that influences laypersons’ action-taking capabilities during an emergency is the level of their self-efficacy [28, 29].

Self-efficacy scales have been developed to measure people’s emotional readiness to face emergencies [30–32]. The main limitations of the existing scales are the variations in the validation processes and the lack of a congruent and domain-specific scale for the general adult population without a healthcare background. Our study aimed to develop a valid and unidimensional self-efficacy scale to capture laypersons’ beliefs in their abilities to help in emergencies and accidents. A deductive method is used, where a systematic assessment of the existing scales is first performed, after which the validation on an adaptation of a scale is performed [33, 34].

### Conceptualization of self-efficacy

According to social cognitive theory, an individual’s behavior depends on continuous interactions between cognitive, behavioral, and environmental factors [35–37]. In this context, *self-efficacy* refers to the belief in an individual’s capability to successfully perform a particular task [36, 38]. Self-efficacy beliefs can vary based on the situation, and the actions needed to perform difficult tasks are more likely to be carried out with a high level of self-efficacy [35, 36]. Bandura [39] considered self-efficacy domain and task-specific, although beliefs about self-efficacy may appear more general in certain situations [36]. Though this more general approach has gained acceptance [40], many studies have consistently argued that the measurement of self-efficacy beliefs should be based on domain-specific evaluations [36, 38, 39].

The concept of self-efficacy has provided a compelling explanation of how and why behavior can change, and it has been studied extensively in various contexts [38]. The level of self-efficacy has been used, for example, for predicting an individual’s health or safety behavior, job performance, initial disaster management, and educational achievement [41–47]. During the past ten years, an increasing number of studies has evaluated the role of self-efficacy on the readiness to help in emergencies [32, 48–50].

In conclusion, self-efficacy is a key motivational construct influencing an individual’s choices, efforts, and persistence, thereby predicting subsequent motivational outcomes and achievements [51]. The role of self-efficacy is increasingly essential for our understanding of how to analyze and achieve helping behavior, which means that we should be able to analyze the self-efficacy level rigorously. For instance, even if many empirical studies conclude that first aid training has a positive effect on the level of self-efficacy, no consistent evaluation method to assess the self-efficacy after first aid training exists [23].

## Systematic assessment of existing first aid self-efficacy scales

**Fig. 1:**
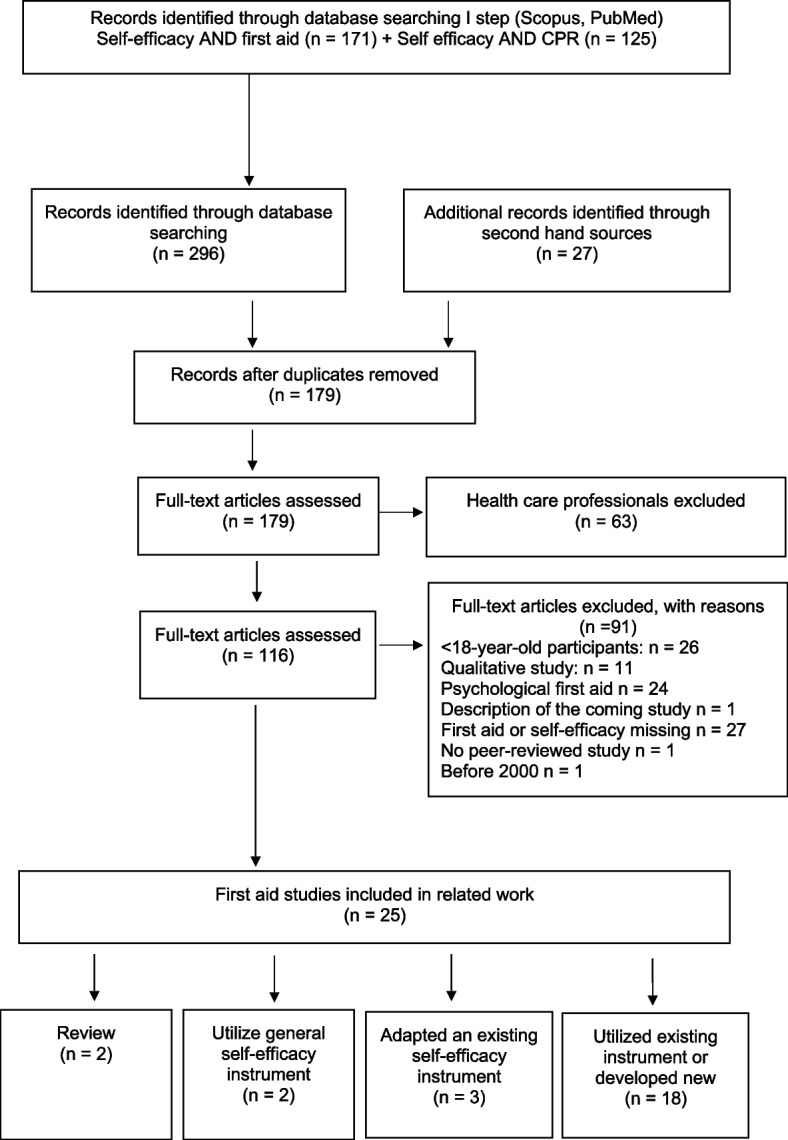
Phases of the systematic identification of existing first aid self-efficacy scales

To properly position our work, we systematically assessed the existing literature about first aid self-efficacy scales (see Fig. 1). The assessment of the existing scales followed the search and selection principles outlined in the PRISMA (Preferred Reporting Items for Systematic Reviews and Meta-Analyses) 2021 guidelines [52]. Scopus and Pubmed were used as the search databases. Because the ’International Journal of First Aid Education’ is not indexed in these two databases but potentially contains relevant information, it was included as a second-hand source. The inclusion criteria were the following:

1. Participants were 18 years or older without a health education background.
2. Studies were written in English and published between 2000 and 2023.
3. Studies followed the guidelines on first aid published by the American Heart Association (AHA) or the European Resuscitation Council (ERC).
4. The evaluation of first aid self-efficacy was based on a self-reported instrument that is repeatable and provides a quantitative outcome.

The Database search started on April 2023 and ended on May 2023; the studies included were published between January 1, 2000, and April 31, 2023. The following two search strings, combined with OR operator, were used in both Scopus and PubMed: (1) ‘first aid’ AND ‘self-efficacy’, and (2) ‘CPR’ AND ‘self-efficacy’. After the first search, duplicates were excluded. The number of studies meeting the search criteria in this first step of the selection process was 179. After all studies that included healthcare professionals were excluded, 116 studies were screened, and 91 articles were excluded according to the inclusion criteria (see Fig. 1). After evaluating the full-text articles and the second application of the inclusion criteria, the number of included studies was two systematic reviews and 23 primary studies (Table 1). The description of the overall selection process is given in Fig. 1.

**Table 1:** Research that utilized an existing instrument or developed a new one for measuring self-efficacy in a first-aid context

Source	Domain	Dims	Items	Validation details
[32]	Resuscitation Self-Efficacy Scale (RSES)	4	17	*n* = 509 (nurses in non-critical care from Korea), CVI, EFA, coefficient $\alpha$
[53]	Self-Efficacy of First Aid in Unintentional Injury at Home	2	37	*n* = 455 (parents from Taiwan), face validity by an expert panel, coefficient $\alpha$, KR20
[31]	Basic Resuscitation Skills Self-Efficacy (BRS-SES)	3	18	*n* = 768 (nursing students from Spain and UK), readability, CVI, discriminant validity, criterion validity, PCA, known-group analysis, coefficient $\alpha$
[54]	School Personnel Self-Efficacy Food Allergy and Anaphylaxis Questionnaire (S.PER.SE.FAAQ)	2	8	*n* = 440 (school personnel from Italy), EFA, coefficient $\alpha$
[55]	Extreme Conditions First Aid Confidence Scale (EC-FACS)	1	9	*n* = 83 (expeditioners from Australia), EFA, parallel analysis, inter-item correlation, coefficient $\alpha$
[56]	Remote First Aid Self-Efficacy Scale (RFA SES)	1	30	*n* = 1554+571 (wilderness medical training alumni and students from the US and Canada), PCA, inter-item correlation, test-retest reliability, concurrent validity

Our literature search identified two reviews on CPR training: one included two self-efficacy scales among airline cabin crew members [22], and the other reported four scales used with adult laypersons [57]. However, neither review evaluated the psychometric properties or validity of these measures.

The set of the 23 included primary studies can be divided into three categories: 1) research that utilized a general self-efficacy instrument, 2) research that adapted an existing self-efficacy instrument, and 3) research that utilized an existing instrument or developed a new one for measuring self-efficacy in the first-aid context. In the two studies [58, 59] an existing scale (New General Self-efficacy Scale) that is not targeted to a domain-specific first-aid context was used for measuring self-efficacy. However, as explained in “Conceptualization of self-efficacy” section, self-efficacy is a domain-specific perception, and it is unclear how a general self-efficacy scale can reflect this specificity [38, 60]. Furthermore, ([40], p. 138) pointed out that if the items of a self-efficacy scale “do not refer to a specific situation or task, they leave room for interpretation by the respondent”.

The second category of research identified in the review adapted a general or a domain-specific self-efficacy measure in the context of first aid [48, 61, 62]. These studies did not provide evidence of the adaptation process or psychometric properties beyond the consistency measured using coefficient $\alpha$. However, it is crucial to report adaptation details, examine the psychometric properties of the adapted scale, and provide supporting validity evidence because it is not guaranteed that the adapted version of an existing scale restores the original measurement properties [63, 64].

The third research category utilized an existing instrument or developed a new one (Table 2). Polloni et al. [65] examined school personnel’s self-efficacy in managing food allergy and anaphylaxis using a validated School Personnel Self-Efficacy Food Allergy and Anaphylaxis Questionnaire (S.PER.SE.FAAQ) [54]. Ho et al. [49] examined domestic helpers’ self-efficacy for emergency management for children using a subscale of the Self-Efficacy of First Aid in Unintentional Injury at Home instrument [53]. Yu and Liang [66] examined the first-aid self-efficacy of airline cabin crew, and [67] explored the impact of immersive virtual reality cardiopulmonary resuscitation training on kindergarten teachers’ self-efficacy. The two aforementioned studies utilized the Basic Resuscitation Skills Self-Efficacy scale (BRS-SES), which showed promising psychometric properties; however, it was developed for nursing students [31]. In addition, [66] used the Resuscitation Self-Efficacy Scale (RSES) developed for nurses [32]. D’Angelo et al. [56] developed and validated the Remote First Aid Self-Efficacy Scale using parallel analysis for dimensionality estimation and analyzed concurrent and test-retest reliability. Wallace et al. [55] utilized the Extreme Conditions First Aid Confidence Scale, which was assessed using factor analysis and Cronbach’s $\alpha$. Schumann et al. and Shafer et al. [68, 69] utilized a new measure developed for the study and reported reliability. Yoon et al. [70] reported a mediation model derivation and multiple logistic regression analysis of a single-item measure to operationalize self-efficacy. Some studies did not provide references or evidence on psychometric properties or validity (i.e., [50, 71, 72]), and the following two studies used one item from secondary data concerning CPR self-efficacy [73, 74]. Buckler et al. [75] used secondary data but did not disclose how self-efficacy was operationalized. The lack of details on operationalizing self-efficacy also concerned [27].

**Table 2:** The Finnish items and Likert options of the first aid self-efficacy scale

Item	Argument
Item 1	Pystyisin auttamaan useimmissa ensiaputilanteissa
Item 2	Olen varma, että suoriutuisin haastavista ensiaputilanteista alusta loppuun asti
Item 3	Yleisesti ottaen pystyn mielestäni saamaan aikaan tuloksia ensiaputilanteissa
Item 4	Uskon onnistuvani missä tahansa ensiaputilanteessa
Item 5	Pystyisin auttamaan onnistuneesti monissa ensiaputilanteissa
Item 6	Luotan siihen, että pystyn toimimaan epäröimättä monissa ensiaputilanteissa
Item 7	Verrattuna muihin ihmisiin hallitsen erittäin hyvin useimmat ensiaputilanteet
Item 8	Pystyn suoriutumaan melko hyvin tiukoissakin ensiaputilanteissa

Likert options: 1 = vahvasti eri mieltä, 2 = eri mieltä, 3 = ei samaa eikä eri mieltä, 4 = samaa mieltä, 5 = vahvasti samaa mieltä

The level of self-efficacy is known to be an important factor in predicting laypersons’ actions during emergencies. However, as summarized in Table 2, only in a few studies a validated instrument for measuring the level of self-efficacy in the first-aid context has been depicted. Moreover, most of the proposed scales include multiple dimensions and a large set of items. Details of their validation processes vary. In summary, to analyze and compare different factors that affect laypersons’ willingness and ability to help during emergencies, a need for a simple, unidimensional self-efficacy scale that is congruent, consistent, and properly validated was concluded as a result of the systematic assessment of the existing scales.

## Materials and methods

**Fig. 2:**
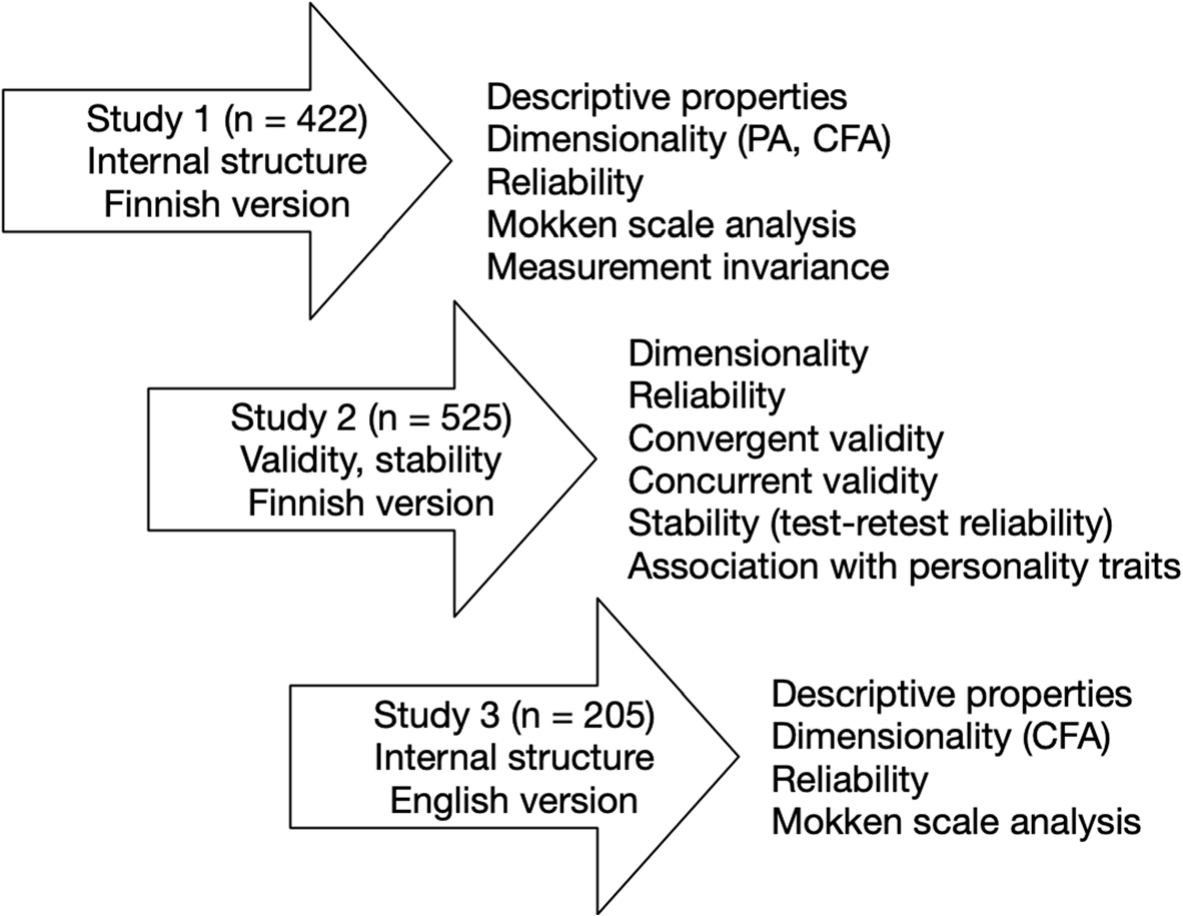
Psychometric properties were examined using three independent samples (in total, *n* = 1152)

The main aim of this research was to construct and validate a new first aid self-efficacy scale. The overall validation process included, firstly, the construction and analysis of the Finnish version of the scale and, secondly, the translation and analysis of the English version to extend the sampled target population. Three independent samples were collected and analyzed for the purpose (Fig. 2), for which we refer in the following as Study 1 (initial psychometric properties of the Finnish version), Study 2 (obtaining validity and stability evidence), and Study 3 (initial psychometric properties of the English version).

### Constructing the first aid self-efficacy scale

The scale construction involved the adaptation of a general self-efficacy instrument into a domain-specific instrument as has been done in the prior literature (e.g., [76–83]). The scale adapted from and translated into Finnish was the New General Self-Efficacy (NGSE) Scale [35, 84], for which permission was asked and received from the principal author. The NGSE scale was chosen as the starting point for the adaptation because it has been shown to display desirable item properties [85]. The first translation of the NGSE scale was done together by an expert in first aid training (MS) and an expert in psychometric analysis (VH). Then, a bi-lingual language professional, speaking both English and Finnish as first languages, finalized the translation. After this, the NGSE scale was adapted to reflect first-aid situations. The Finnish version of the first aid self-efficacy scale was used in Study 1 and Study 2. For Study 3, the Finnish scale was translated to English by the same bi-lingual language professional as previously in the process and evaluated by the authors (MS and VH). This final English translation was evaluated by a British English teacher. The senior author (TK) evaluated, commented on, and concluded all the steps in the adaptation and translation process. The scale items and response categories in Finnish and English are presented in Tables 2 and 3.

**Table 3:** The English items and Likert options of the first aid self-efficacy scale

Item	Argument
Item 1	I would be able to help in most first aid situations
Item 2	I am sure that I would be able to handle challenging first aid situations from start to finish
Item 3	Generally speaking, I think that I can create results in first aid situations
Item 4	I believe I can succeed in any first aid situation
Item 5	I would be able to successfully provide help in many first aid situations
Item 6	I am confident that I can provide help without hesitation in many first aid situations
Item 7	In comparison to other people, I can handle most first aid situations very well
Item 8	I am able to perform quite well even in challenging first aid situations

Likert options: 1 = strongly disagree, 2 = disagree, 3 = neither agree nor disagree, 4 = agree, 5 = strongly agree

### Psychometric protocol

The psychometric analysis of the first-aid self-efficacy scale involved a systematic approach to establish its reliability and validity in measuring self-efficacy in first aid situations (Fig. 2). The analytics approach involved methods from classical test theory (CTT), latent variable analysis, and nonparametric item response theory (e.g., [86]). The scale analysis was conducted utilizing a diverse sample of 1152 participants.

Study 1 involved examining psychometric properties of the scale, including item responses, dimensionality using parallel analysis (PA-PCA) [87] and confirmatory factor analysis (CFA) [88], reliability in the context of CTT using coefficient $\alpha$ [89], scalability in the context of IRT utilizing Mokken scale analysis (MSA) [90–92], and measurement invariance in terms of gender [93, 94]. We used a scalogram to visually represent the variation in respondents’ response patterns [86]. In the scalogram, respondents were arranged based on their total score $X_+$, while the scale items were ordered by their item sum scores. Consequently, colors indicating high levels of agreeableness were concentrated in the top right corner of the figure, whereas colors indicating high levels of disagreeableness were concentrated in the lower left corner.

For PA-PCA, we employed a nonparametric approach to parallel analysis using column permutations (500 random data sets), polychoric correlations, and quantile thresholds set at 50% (median, PA-PCA-m) and 95% (PA-PCA-95) [95, 96]. Minimum average partial (MAP) indicates the optimal number of factors or components to retain in an analysis by identifying the point at which the average partial correlation among variables-after sequentially partialing out extracted factors-is minimized [97]. The CFA model was estimated using polychoric correlation [98] and the robust diagonally weighted least squares (DWLS) method, with test statistics adjusted for mean and variance (also known as the scale-shifted approach or WLSMV [99]), which is recommended for analyzing ordinal data [100–103]. The literature provides various recommendations for threshold values and combination rules to ensure satisfactory CFA model performance: an RMSEA $\le$ 0.05 indicates a close fit, RMSEA between 0.05 and 0.08 indicates a fair fit, RMSEA between 0.08 and 0.10 indicates a mediocre fit [104], and a combination of TLI > 0.95 or CFI > 0.95 and SRMR < 0.09 suggests an acceptable fit [105].

Scalability in MSA relies on the homogeneity coefficient H [92, 106], also known as the scalability coefficient [90]. Existing scales can be assessed using the inter-item coefficients $H_{jk}$, individual item coefficients $H_j$, and the overall scale coefficient *H* (Mokken, 1971, 1997). A higher $H_j$ indicates better item discrimination, whereas values near zero show poor discrimination regarding the latent variable [90, 92]. Therefore, a typical method to determine item inclusion in a scale is to set a threshold value c, ensuring $H_j> c$ for all items. The minimum threshold traditionally used for item inclusion is $H_j> 0.30$, with items below this threshold considered unscalable [90, 92]. For classifying complete scales, 0.30 $\le$ H < 0.40 indicates a weak Mokken scale, 0.40 $\le$ H < 0.50 indicates a medium scale, and H $\ge$ 0.50 indicates a strong scale ([92], p. 185). Mokken scale indicates that the scale can be used to create composite scores and order respondents by the latent trait [107].

Study 2 examined validity evidence by establishing associations with similar measures, first aid knowledge, professionality, and first aid course completion. Moreover, the study established the stability of the first-aid self-efficacy scale in terms of test-retest validity [108, 109] and examined associations between the developed scale and Big-Five personality traits using the Ten Item Personality Inventory [110, 111]. Study 3 involved examining psychometric properties of the English version of the first-aid self-efficacy scale, including dimensionality, reliability, and scalability analysis.

**Fig. 3:**
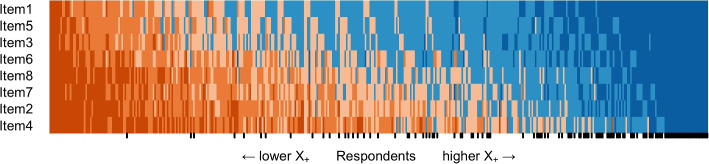
The scalogram of all responses (*n* = 422). Respondents are ordered by total score $X_+$, and items are arranged by invariant item ordering. Ticks in the x-axis denote the responses of healthcare professionals

**Fig. 4:**
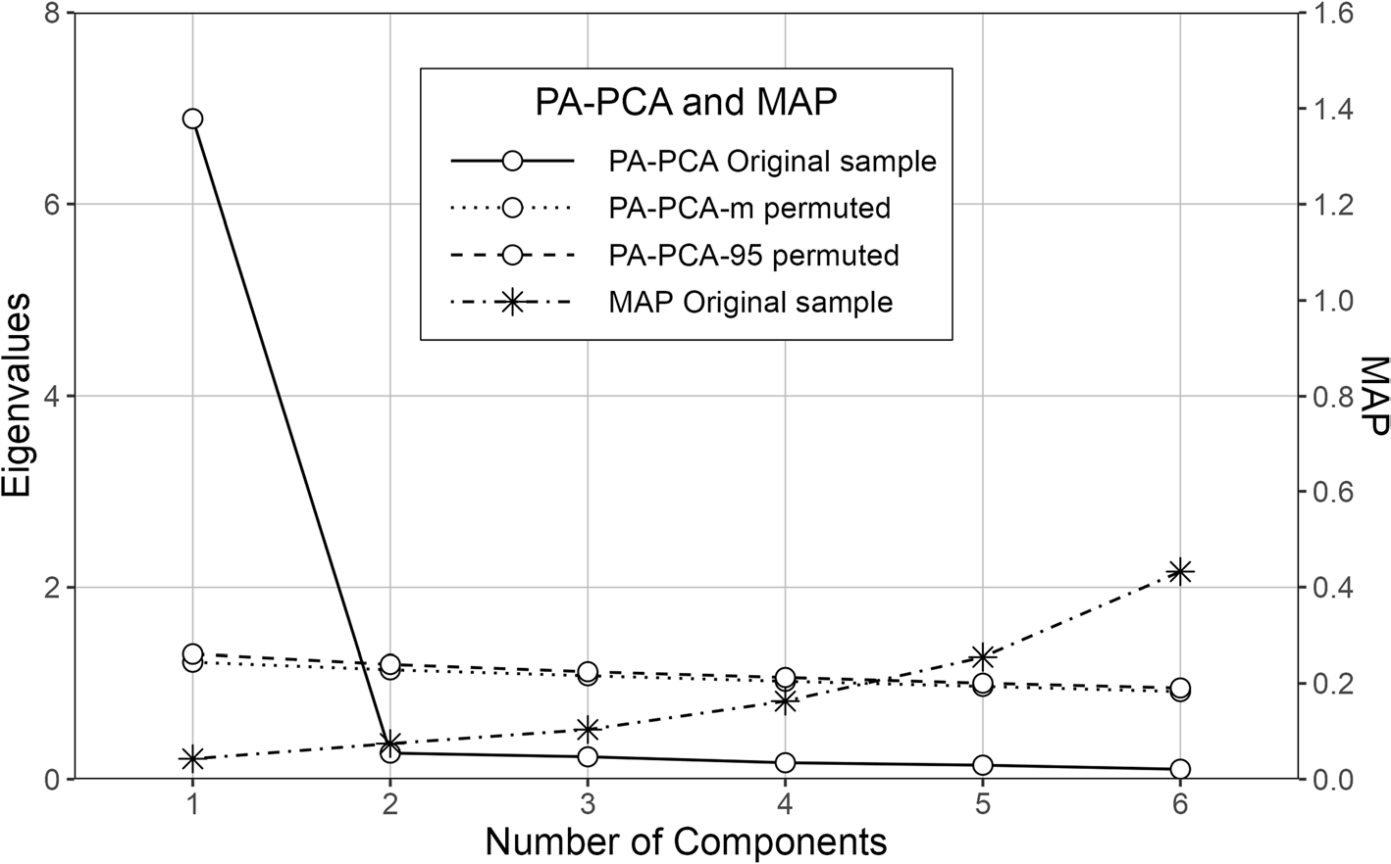
Both MAP and parallel analysis using PCA supported a structure with one component

**Table 4:** Properties of the items ordered by the invariant item ordering (descending order by item difficulty, the first Item 1 easiest to agree with)

Item	M	SD	Skew $G_1$	Kurtosis $G_2$	$r_s$	$H_{ij}$	$H_i$
Item 1	3.74	1.08	−0.71	0.20	0.74–0.81	0.81–0.88	0.84
Item 5	3.63	1.10	−0.58	−0.49	0.70–0.83	0.79–0.87	0.83
Item 3	3.61	1.11	−0.60	−0.37	0.74–0.82	0.80–0.85	0.83
Item 6	3.37	1.24	−0.32	−0.91	0.66–0.83	0.73–0.89	0.81
Item 8	3.07	1.29	−0.12	−1.07	0.77–0.83	0.84–0.89	0.85
Item 7	3.01	1.28	0	−1.04	0.74–0.82	0.80–0.86	0.83
Item 2	2.85	1.31	0.18	−1.06	0.76–0.82	0.82–0.88	0.84
Item 4	2.53	1.27	0.40	−0.93	0.68–0.78	0.73–0.85	0.81

## Results

### Study 1

The primary aim of Study 1 was to evaluate the internal structure of the Finnish version of the scale for measuring first-aid self-efficacy. The key properties of the scale were examined through rigorous psychometric analysis, including item responses, dimensionality, reliability, scalability, and measurement invariance regarding gender.

#### Data collection and participants

Data were collected through an anonymous online questionnaire distributed on social media and professional networks (e.g., via email, instant messaging, and intranet). Participation was voluntary, and before participation, individuals were informed about the nature and purpose of the study. The questionnaire included items assessing background variables and the primary measurement scale in Finnish. In the complete sample of Study 1 (*N* = 422), 63% were women, 35% were men, and 2% identified themselves as nonbinary. Most were laypeople, and healthcare professionals made up 28% of the sample. The average age of participants was 39.4 years (SD = 13.8, Mdn = 38, min = 17, max = 77). In terms of education, 71% of participants had completed their education at the International Standard Classification of Education (ISCED) levels 6–7, while 29% had completed their education at levels 2–4.

#### Descriptive properties

All categories of each item received responses. The lowest number of responses were in the Totally disagree categories of Item 1 (3.3%) and Item 5 (3.8%). In general, Item 1 was the easiest item for respondents to agree with, and Item 4 was the most difficult item to agree with. As can be expected, healthcare professionals tended to agree with the items scoring higher total scores (Fig. 3).

#### Dimensionality and reliability

Parallel analysis and the smallest MAP suggested a unidimensional structure for the scale (Fig. 4). Based on CFA, the unidimensional factor structure demonstrated a strong fit, showing sufficient values for key fit indices: $\chi ^2 (20; n = 422) = 97.371$, $p < .001$; CFI = .997; TLI = .996; RMSEA = .096, 95%CI [.077, .115], $p_{close} < .001$; SRMR = .019. Inter-item Spearman’s rank correlation coefficients $r_s$ (Table 4) ranged from .66–.83, indicating high internal consistency. The lower bound to the reliability as measured using coefficient $\alpha$ suggested strong reliability ($\alpha$ = .96, 95%CI [.96, .97]). In general, the scale demonstrated the expected structural validity in terms of dimensionality as assessed using the factor analysis framework, and it showed robust reliability in the framework of CTT.

#### Scalability and invariant item ordering

The latent monotonicity assumption was met because the inspection of the ISRFs and IRFs of the items and the investigation of monotonicity [112] showed that there were no significant violations of manifest monotonicity using minimum rest score group sizes of N/5, N/10, and N/20. Procedure CA [113] indicated local independence. Coefficients of homogeneity (Table 4) for the inter-item pairs $H_{ij}$ ranged from .73–.89. For individual items, coefficients of homogeneity $H_i$ ranged from .81–.85, exceeding the traditional threshold value of c = .30. Thus, the model met the assumptions of the monotone homogeneity model, indicating that the scale formed a strong Mokken scale ($H = .83$). Furthermore, the restscore [112] showed that there were no significant violations of nonintersection. Thus, the model met the assumptions of the double monotonicity model ($H^T = .57$), indicating that items may also be ordered with high accuracy with respect to the latent variable [112, 114]. Table 4 depicts the invariant item ordering of the scale, which reflects the ordering by the item’s mean value.

#### Measurement invariance

Measurement invariance across gender was assessed by evaluating multi-group CFA models [93] for women (*n* = 264) and men (*n* = 149). A sequence of nested model comparisons using the scaled chi-squared difference test [115] was conducted to evaluate the measurement invariance models. The model constraining thresholds and loadings to be equal between groups showed the best fit ($\chi ^2 (63; n = 264/149) = 133.073$, *p* = .000; CFI = .998; TLI = .998; RMSEA = .074, 95%CI [.056, .091], $p_{close} = .015$; SRMR = .021), suggesting a strong measurement invariance between men and women.

### Study 2

The primary aim of Study 2 was to accumulate additional validity evidence for the Finnish version of the scale, focusing on assessing its properties across diverse populations. The study utilized various methodologies to establish convergent and concurrent validity, including comparisons with the New General Self-Efficacy Scale (NGSE) and the Adult Basic Life Support (BLS) measure. Additionally, this study examined the scale’s associations with personality traits and its consistency over time, as evidenced by its test-retest reliability.

#### Data collection and participants

Data were collected through an anonymous online questionnaire distributed on social media and professional networks (e.g., via email, instant messaging, and intranet). Participation was voluntary, and before participation, individuals were informed about the nature and purpose of the study. The questionnaire included items assessing background variables, a knowledge test, and the primary measurement scale in Finnish. In the complete sample of Study 2 (*N* = 525), 78% were women, 20% were men, and 2% identified themselves as nonbinary. Most were laypeople, and healthcare professionals constituted 29% of the sample. The average age of participants was 41 years (SD = 13.2, Mdn = 40, min = 16, max = 76). Regarding education, 70% of participants had completed their education at the ISCED levels 6–7, while 30% had completed their education at levels 2–4. A sub-sample of the same respondents (*n* = 131) answered the questionnaire the second time after four weeks for estimating scale stability.

#### Measures

The New General Self-efficacy Scale (NGSE) was used to evaluate convergent validity with the adapted scale. General self-efficacy has been associated with conscientiousness and psychological health [116]. In addition, persons reporting high general self-efficacy can be successful in various domains [117]. Pulkka and Budlong [118] utilized a Finnish version of NGSE but did not provide structural details of the scale. Based on CFA, the NGSE in this study showed sufficient fit for the unidimensional model, $\chi ^2 (20; n = 525) = 64.451$, *p* = .000; CFI = .993; TLI = .989; RMSEA = .076, 95%CI [.057, .096], $p_{close} = .013$; SRMR = .025. The lower bound to the reliability was high ($\alpha$ = .90, 95%CI [.88, .92]). AISP indicated a unidimensional scale at the level of c = .55, and the scale formed a strong Mokken scale (H = .61).

The self-confidence in performing the Adult Basic Life Support (BLS) measure [30] was used to assess the convergent validity of the first-aid self-efficacy scale with a similar construct. In this study, BLS showed sufficient fit for the unidimensional model, $\chi ^2 (14; n = 525) = 52.859$, *p* = .000; CFI = .949; TLI = .924; RMSEA = .073, 95%CI [.053, .094], $p_{close} = .033$; SRMR = .031, and high reliability, $\alpha$ = .92, 95%CI [.91, .93].

The level of first aid knowledge was measured with six multiple-choice questions about different first aid subjects (traffic accident, stroke, low blood sugar, CPR, the use of a defibrillator, and unconsciousness). The questionnaire (Appendix A) was based on the international guidelines about life-saving skills [18] and was provided in Finnish to the participants in the same questionnaire as the first aid self-efficacy scale. The participants were asked to answer as honestly as possible, using only their knowledge and no external sources. Some of the answers had more than one correct option, of which the participants also were informed.

**Table 5:** Properties of the items according to invariant item ordering

Item	M	SD	Skew $G_1$	Kurtosis $G_2$	$r_s$	$H_{ij}$	$H_i$
Item1	3.97	1.01	−0.97	0.36	0.68–0.79	0.78–0.90	0.82
Item5	3.87	0.99	−0.98	0.65	0.64–0.78	0.76–0.85	0.80
Item3	3.85	0.98	−0.96	0.73	0.65–0.74	0.74–0.82	0.79
Item6	3.58	1.16	−0.56	−0.61	0.68–0.81	0.74–0.87	0.81
Item8	3.28	1.19	−0.36	−0.80	0.74–0.82	0.79–0.87	0.84
Item7	3.26	1.16	−0.20	−0.80	0.71–0.81	0.79–0.84	0.81
Item2	3.14	1.26	−0.20	−1.10	0.71–0.83	0.82–0.90	0.86
Item4	2.78	1.19	0.16	−0.98	0.64–0.83	0.76–0.90	0.82

**Table 6:** Pearson product-moment correlation coefficients between the first-aid self-efficacy scale and measures used to examine validity evidence

	KNOW	NGSE	BLS$$^{1)}$$
Developed scale	$$.58^{***}$$	$$.43^{***}$$	$$.83^{***}$$
KNOW		$$.26^{***}$$	$$.68^{***}$$
NGSE			$$.37^{***}$$

^1^[30]

***p < .001

**Table 7:** Total first-aid self-efficacy score and statistically significant differences between groups using the Welch two sample two-sided t-test

	N	M	Sd	Mdn	Min	Max	Skew $G_1$	Kurtosis $G_2$
All	525	27.7	7.9	29	8	40	−0.44	−0.43
Non-professionals	374	$$25.4^{1)}$$	7.6	26	8	40	−0.25	−0.53
Professionals	151	33.5$$^{1)}$$	5.0	33	21	40	−0.41	−0.58
Non-professionals, men	83	28.8$$^{2)}$$	7.1	30	8	40	−0.37	−0.28
Non-professionals, women	279	24.4$$^{2)}$$	7.6	25	8	40	−0.20	−0.61

$$^{1)}$$ t(420.6) = −14.30, $$p < .001$$; $$^{2)}$$ t(142.6) = −4.97, $$p < .001$$

**Fig. 5:**
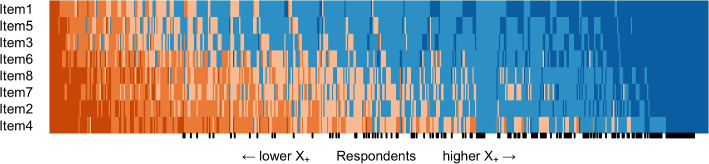
The scalogram of all responses (*n* = 525). Respondents are ordered by total score $X_+$, and items are arranged according to invariant item ordering. Ticks in the x-axis denote the responses of healthcare professionals

#### Dimensionality and reliability

In Study 2, the item properties (Table 5) were similar to the results obtained in Study 1 (Table 4), and the scalogram showed a similar structure (Fig. 5). Additionally, the scale showed a unidimensional structure with a sufficient fit ($\chi ^2 (17; n = 525) = 66.077$, *p* = .000; CFI = .998; TLI = .997; RMSEA = .074, 95%CI [.056, .094], $p_{close} = .016$; SRMR = .015) after adding three correlated residuals between three similarly worded item pairs: Item3–Item5, Item2–Item4, and Item1–Item5. Moreover, strong measurement invariance by gender was confirmed ($\chi ^2 (63; n = 406/103) = 174.015$, *p* = .000; CFI = .996; TLI = .997; RMSEA = .083, 95%CI [.069, .098], $p_{close} = .000$; SRMR = .027). The scale showed strong reliability ($\alpha = .96$, 95%CI [.95, .96]) and again formed a strong Mokken scale ($H = .82$) showing double monotonicity ($H^T$ = .64) with only two significant violations of invariant item ordering in items 2 and 7 (Tables 6 and 7).

#### Convergent validity

Associations of the first aid self-efficacy scale with other closely related constructs can be interpreted as evidence of convergent validity (Table 5). Notably, the developed scale showed a strong positive convergent association (i.e., [119]) with self-confidence in performing basic life support using the BLS measure by [30]. Conversely, the general self efficacy measure NGSE showed moderate associations (i.e., [120]) with the first-aid self-efficacy scale and BLS, suggesting that a general measure does not fully capture a domain-specific construct. The result suggests that the developed scale especially captures the context of everyday emergency situations while embracing the self-efficacy perspective. Because BLS showed an increase after two-hour training [30], a high convergent validity between the BLS measure and first-aid self-efficacy scale suggests that a first-aid self-efficacy scale could also be utilized in educational settings.

#### Concurrent validity

**Fig. 6:**
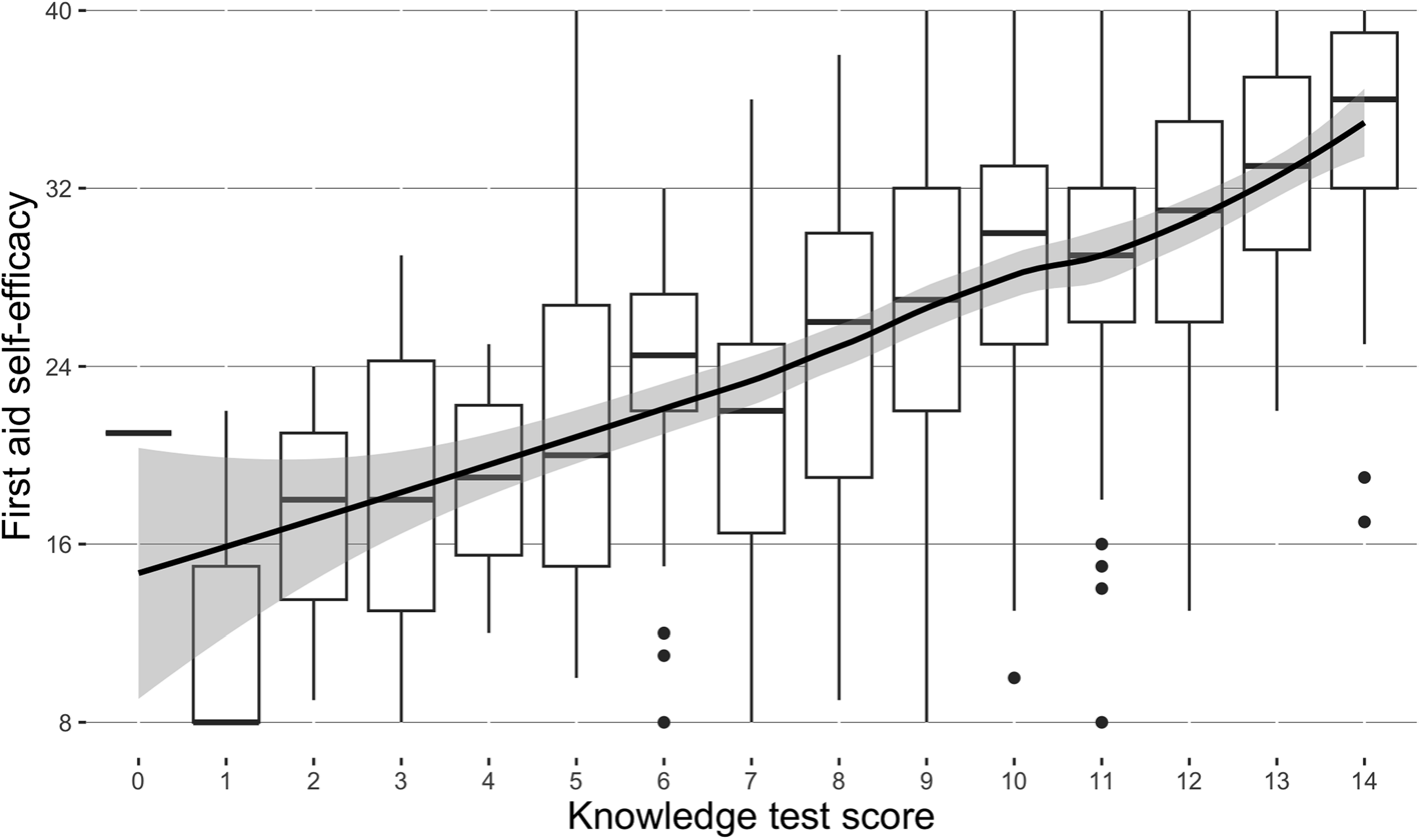
Total score of first-aid self-efficacy by knowledge test score

**Fig. 7:**
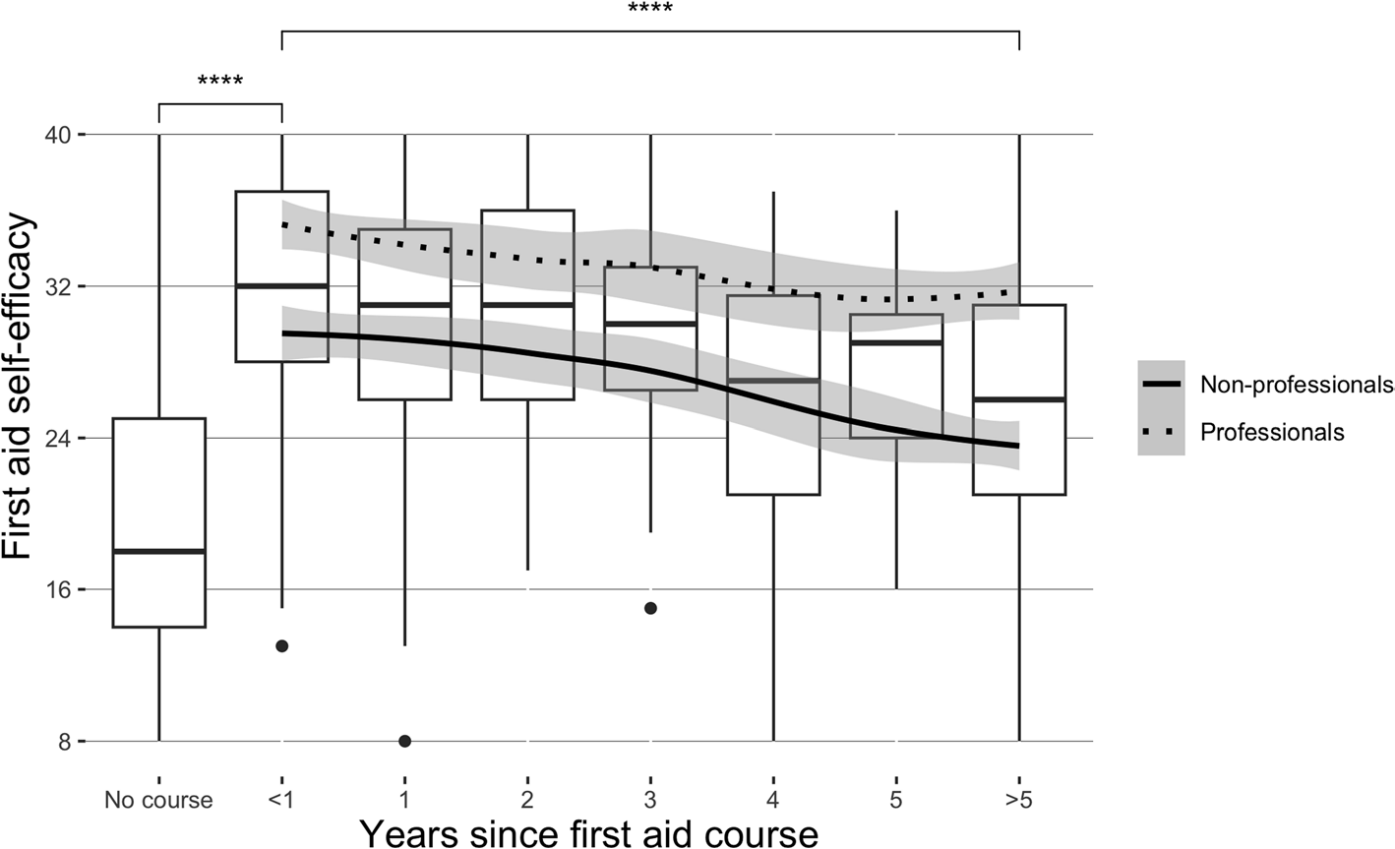
First aid self-efficacy sum score by years since attending the last first aid course for the complete sample and lines for professionals and non-professionals (laypeople). Statistical significance is estimated using the Welch two-sample t-test for just after completing a first aid course and after five years

A high correlation between the BLS measure and first-aid self-efficacy scale and the statistically significant difference in first-aid self-efficacy between healthcare professionals and non-professionals suggested evidence of concurrent validity (Table 5). As expected, healthcare professionals showed higher first-aid self-efficacy scores than non-professionals. Interestingly, men showed slightly higher scores than women in the subgroup of non-professionals; however, the result should be interpreted with caution because of the unbalanced sample in terms of gender. For the complete sample, Fig. 6 shows for the complete sample the total first-aid self-efficacy score by knowledge test score (M = 9.8, SD = 3.1, Mdn = 10, Min = 0, Max = 14). The result suggests that first-aid self-efficacy is associated with a knowledge of first aid procedures and situations. For the complete sample, Fig. 7 depicts the first-aid self-efficacy sum score by years since attending the last first aid course. Taking a first aid course improves first-aid self-efficacy significantly based on the Welch two-sample t-test (t(108.7) = −11.5, *p* < .001), after which it starts to decay slowly. Notably, five years after taking the first aid course, the self-efficacy in first aid was statistically significantly lower than just after the course (t(256.7) = 7.2, *p* < .001). Both findings above add to the evidence of concurrent validity and highlight the importance of first aid knowledge and regular first aid courses.

#### Stability

Test-retest reliability indicates that a test is consistent and stable in its measurements over time and, thus, has the potential to predict future behavior ([109], p. 255). A total of 131 respondents answered the scale for the second time after four weeks. Excellent test-retest reliability (i.e., [108]) of the scale, as measured using the Pearson product-moment correlation coefficient between total scores, *r* = .94, and the intraclass correlation coefficient, ICC = .93, 95% CI [.90–.95], indicated the temporal stability of the scale after four weeks.

#### Association with personality traits

**Fig. 8:**
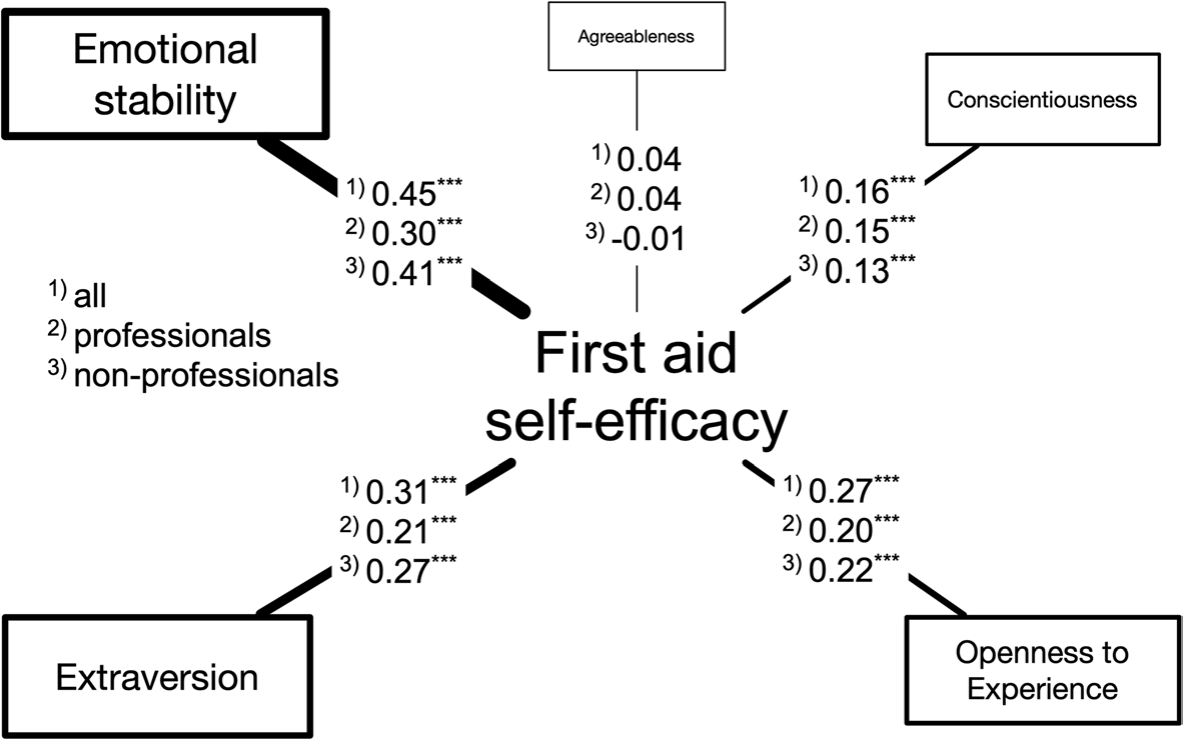
Associations of first-aid self-efficacy (i.e., Pearson product-moment correlation coefficients) with Big Five personality traits measured using TIPI for the whole sample and subsets of healthcare professionals and non-professionals

In the context of the associations between first-aid self-efficacy and various personality traits measured using the Ten Item Personality Inventory (TIPI) in the Big Five framework [110, 111], the study showed distinct correlations across respondent categories: all participants, healthcare professionals, and non-professionals (Fig. 8). Emotional stability exhibited a robust positive association with first-aid self-efficacy scores, with the highest correlation seen in the general sample (*r* = .45), followed by non-professionals (*r* = .41) and professionals (*r* = .30). Extraversion and openness to experiences also showed significant positive associations with the self-efficacy of first aid, though to a lesser extent compared to emotional stability. Conversely, conscientiousness and agreeableness appeared to have a small to negligible link with first-aid self-efficacy across all groups. The results for emotional stability, extraversion, openness to experiences, and agreeableness in this study aligned well with a meta-analysis that evaluated associations between general self-efficacy and Big Five personality traits [121]. Interestingly, conscientiousness in this study showed a lower association than in the meta-analysis.

### Study 3

The aim of Study 3 was to evaluate the internal structure of the English version of the scale. The key properties of the scale were examined through psychometric analysis similar to Study 1, including item responses, dimensionality, reliability, and scalability.

#### Data collection and participants

Data were collected through an anonymous online questionnaire distributed on social media and professional networks (e.g., via email, instant messaging, and intranet). Participation was voluntary, and before participation, individuals were informed about the nature and purpose of the study. The questionnaire included items assessing background variables and the primary measurement scale in English. In the complete sample of Study 3 (N = 205), 72% were women, 27% were men, and 1% identified as nonbinary. Most were laypeople, and healthcare professionals made up 17% of the sample. The average age of participants was 39.5 years (SD = 14.3, Mdn = 40, min = 17, max = 79). In terms of education, 71% of participants had completed their education at the International Standard Classification of Education (ISCED) levels 6–7, while 29% had completed their education at levels 2–4.

#### Descriptive properties

**Fig. 9:**
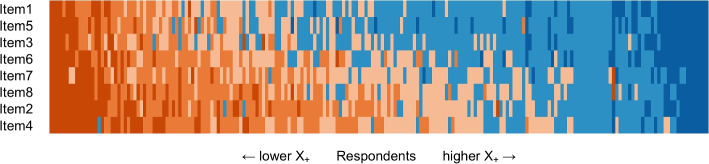
The scalogram of all responses (*n* = 205). Respondents are ordered by total score $X_+$, and items are ordered by invariant item ordering

The descriptive properties of the English version of the first-aid self-efficacy scale were comparable to the properties of the Finnish version of the scale in studies 1 and 2 (Table 8). However, item means were slightly lower as the sample consisted of fewer healthcare professionals than in studies 1 and 2. All categories of each item received responses. The lowest number of responses were in the Totally disagree category of Item1 (6.3%) and Totally agree categories of Item4 (6.3%) and Item8 (6.3%), which can be expected as Item1 was the easiest item to agree with, and the other two items were, in general, more difficult to agree with. Again, based on the item means, the ordering of the items in terms of difficulty was the same as found in studies 1 and 2, except items ATI7 and ATI8 switched places. The Scalogram of the English version showed a similar visual structure as in studies 1 and 2 (Fig. 9). The total score was comparable to the sample of non-professionals in Study 2 (M = 25.04, SD = 7.9, Mdn = 26, Min = 8, Max = 40, $G_1 = -0.23$, $G_2 = -0.62$).

#### Dimensionality and reliability

Based on CFA, the unidimensional factor structure of the English version demonstrated excellent values for key fit indices after adding correlated residuals (Item7–Item8, Item1–Item5, Item3–Item5, and Item1–Item3): $\chi ^2 (16; n = 205) = 35.108$, $p = .004$; CFI = .998; TLI = 0.996; RMSEA = .077, 95%CI [.042, .111], $p_{close} < .096$; SRMR = .015. Correlated residuals can be justified based on the similar semantics of the items’ wording. Inter-item Spearman’s rank correlation coefficients $r_s$ (Table 6) ranged from .66–.79. The lower bound to the reliability is measured using coefficient $\alpha$ suggested strong reliability ($\alpha$ = .95, 95%CI [.94, .96]). In general, the scale demonstrated a similar structural validity as in studies 1 and 2 in terms of dimensionality as assessed using the factor analysis framework and showing robust reliability in the framework of CTT.

#### Scalability and invariant item ordering

The inspection of the ISRFs and IRFs of the items and the investigation of monotonicity [112] showed that there were no significant violations of manifest monotonicity using minimum rest score group sizes of N/5, N/10, and N/20, indicating that the latent monotonicity assumption was met. Procedure CA [113] indicated local independence. The coefficients of homogeneity (Table 6) for the inter-item pairs $H_{ij}$ ranged from .71–.85. For individual items, the coefficients of homogeneity $H_i$ ranged from .76–.80, showing slightly lower values than in studies 1 and 2 but still clearly exceeding the traditional threshold value of c = .30. Thus, the nonparametric IRT model of the English version met the assumptions of the monotone homogeneity model, indicating that the scale formed a strong Mokken scale ($H = .78$). Furthermore, the method’s rest score [112] showed that there were only two significant violations of nonintersection. Thus, the model could be considered to meet the assumptions of the double monotonicity model ($H^T = .39$), indicating that items may also be ordered with high accuracy with respect to the latent variable [112, 114]. Table 6 depicts the invariant item ordering of the scale, which reflects the ordering according to the items’s mean value, as was found also in studies 1 and 2.

## General discussion

Our research aimed to develop a consistent and unidimensional first aid self-efficacy scale that can be used to measure the level of self-efficacy and promote laypersons’ actions during emergencies and accidents. For this purpose, the developed scale was detected to be internally consistent and stable, and its psychometric properties were established. The scale reliably reflects the level of people’s self-efficacy when facing challenging emergencies.

**Table 8:** Properties of the items according to invariant item ordering

Item	M	SD	Skew $G_1$	Kurtosis $G_2$	$r_s$	$H_{ij}$	$H_i$
Item1	3.52	1.08	−0.72	−0.12	0.67–0.79	0.76–0.85	0.80
Item5	3.51	1.14	−0.73	−0.32	0.65–0.79	0.73–0.84	0.78
Item3	3.44	1.08	−0.70	−0.17	0.64–0.75	0.71–0.81	0.76
Item6	3.17	1.21	−0.19	−0.99	0.64–0.75	0.71–0.81	0.78
Item7	3.04	1.08	−0.17	−0.61	0.65–0.76	0.73–0.82	0.76
Item8	2.83	1.15	0.01	−0.93	0.66–0.78	0.72–0.82	0.78
Item2	2.78	1.23	0.11	−1.11	0.67–0.78	0.75–0.85	0.79
Item4	2.75	1.16	0.11	−0.89	0.67–0.76	0.72–0.80	0.76

To evaluate human behavior, [122] described the importance of well-designed and validated scales. Though plenty of scales have been adapted and developed to measure a range of social, psychological, and health behaviors and experiences, only a few have concentrated on measuring the effectiveness of different interventions to improve first-aid skills and laypersons’ self-efficacy enabling them to help during emergencies. The developed scale is novel, showing validity evidence, and concentrates specifically on laypersons’ self-efficacy in first aid situations.

Many recommendations and interventions have been launched after 2020 to address the issue of safety behavior globally. For example, the target of EU’s Road Safety Policy Framework 2021–2030 is to reduce the number of deaths to almost zero by 2050 [19]. By 2030 worldwide, the United Nations General Assembly has set a goal of halving the global number of road deaths and injuries [123]. Furthermore, for example, the aim of the worldwide Stop the Bleed^®^ program is to train 200 million people in hemorrhage control in the near future [4, 124]. First aid given by a layperson can be one way to reduce and prevent deaths in our society. By investing in first aid training, we can increase the number of skilled laypersons, enabling us to help during emergencies. However, psychomotor skills and knowledge of first aid do not necessarily translate into helping behavior [125–127].

Laypersons’ medically optimal actions in emergencies have been well-studied and well-defined. Therefore, the natural target of first-aid education has been to increase laypersons’ first-aid knowledge and skills [23]. During the last few years, there has been an increasing interest in the role of emotions in predicting behavior during emergencies [128, 129]. In our systematic identification of existing first aid self-efficacy scales, the role of self-efficacy was evident. Of the found 25 studies, the number published studies after 2016 was 21. Nowadays, in addition to aiming to improve laypersons’ knowledge and skills in first aid training, we should pay attention to self-efficacy and emergency attitudes to enhance and encourage people to be better prepared for emergencies and accidents [4]. The challenge in measuring self-efficacy after first aid training and in self-efficacy studies has been the variations in self-efficacy scales. The novel first-aid self-efficacy scale presented in our research fills this gap, allowing us to measure the effectiveness of first-aid interventions on self-efficacy and promoting immediate responders’ ability to help.

### Limitations and future work

Our research has some limitations that should be taken into consideration. The data was based on self-reported questionnaires, influenced possibly by social expectations, self-reported errors, and unreliable memories. Moreover, questionnaires may introduce acquiescence bias, which refers to the tendency of the respondents to agree (or disagree) with the claims independently on their contents [130]. In contrast, the respondents’ willingness to answer the questionnaire can lead to bias because only those who were motivated and interested answered the questionnaire. Finally, potential respondents were not randomly selected. However, the questionnaire was sent widely and, therefore, reached many of the general population. Future work should examine first aid self-efficacy in educational settings (e.g., the effectiveness of first-aid training) and in simulated experiments (e.g., the actualized propensity to give first aid). Even if the 8-item form of the proposed first aid self-efficacy scale is as simple as the simplest counterpart in Table 2, one could readdress its structural complexity to reduce the number of items while maintaining congruency and consistency.

## Conclusion

The studies presented in this paper showed that the developed 8-item scale is unidimensional, reliable, and valid to measure the level of people’s self-efficacy to face different emergencies and accidents. The scale of first-aid self-efficacy was detected to be internally consistent and stable, and its psychometric properties were established. The level of self-efficacy in the case of facing an emergency was dependent on training and experience. Those with a history of first aid training had a higher self-efficacy level than those without a training history. Healthcare professionals had the highest level of self-efficacy. Furthermore, as time passed after the first aid training, the level of self-efficacy decreased.

Studying the association between self-efficacy and first aid skills could assist us in developing various strategies to improve laypersons’ ability and willingness to help in different emergencies and accidents. The developed scale is a valid tool and could be widely used to measure the level of the self-efficacy of attendants before and after various interventions seeking to add people’s ability to act in different emergencies.

## Supplementary Information

Questionnaire instruments.

## Data Availability

The authors do not have permission to share data as per research permissions.

## References

[1] European Union. Accidents and injuries statistics. 2022. https://ec.europa.eu/eurostat/statistics-explained/index.php?title=Accidents_and_injuries_statistics#Deaths_from_accidents.2C_injuries_and_assault. Accessed 14 May 2024.

[2] Eropean Union. Road safety statistics in the EU. 2022. https://ec.europa.eu/eurostat/statistics-explained/index.php?title=Road_safety_statistics_in_the_EU#The_number_of_persons_killed_in_road_traffic_accidents_increased_in_2021.2C_after_decreasing_continuously_since_2011. Accessed 14 May 2024.

[3] World Health Organization. Status report on road safety 2018: summary. 2018. https://www.who.int/publications/i/item/WHO-NMH-NVI-18.20. Accessed 14 May 2024.

[4] Goralnick E, Ezeibe C, Chaudhary MA, McCarty J, Herrera-Escobar JP, Andriotti T, et al. Defining a Research Agenda for Layperson Prehospital Hemorrhage Control: A Consensus Statement. JAMA Netw Open. 2020;3(7):e209393. 10.1001/jamanetworkopen.2020.9393. 32663307 10.1001/jamanetworkopen.2020.9393

[5] Rhee P, Joseph B, Pandit V, Aziz H, Vercruysse G, Kulvatunyou N, et al. Increasing Trauma Deaths in the United States. Ann Surg. 2014;260(1):13. 10.1097/SLA.0000000000000600. 24651132 10.1097/SLA.0000000000000600

[6] Sánchez-Mangas R, García-Ferrrer A, De Juan A, Arroyo AM. The probability of death in road traffic accidents. How important is a quick medical response? Accid Anal Prev. 2010;42(4):1048–1056. 10.1016/j.aap.2009.12.012. 10.1016/j.aap.2009.12.012 20441812

[7] Oliver GJ, Walter DP, Redmond AD. Prehospital deaths from trauma: Are injuries survivable and do bystanders help? Injury. 2017;48(5):985–91. 28262281 10.1016/j.injury.2017.02.026

[8] Chen KY, Ko YC, Hsieh MJ, Chiang WC, Ma MHM. Interventions to improve the quality of bystander cardiopulmonary resuscitation: A systematic review. PLoS ONE. 2019;14(2):e0211792. 10.1371/journal.pone.0211792. 30759140 10.1371/journal.pone.0211792 PMC6373936

[9] Blom MT, Beesems SG, Homma PCM, Zijlstra JA, Hulleman M, van Hoeijen DA, et al. Improved Survival After Out-of-Hospital Cardiac Arrest and Use of Automated External Defibrillators. Circulation. 2014;130(21):1868–1875. Publisher: American Heart Association. 10.1161/CIRCULATIONAHA.114.010905. 10.1161/CIRCULATIONAHA.114.010905 25399395

[10] Berdowski J, Blom MT, Bardai A, Tan HL, Tijssen JGP, Koster RW. Impact of Onsite or Dispatched Automated External Defibrillator Use on Survival After Out- of-Hospital Cardiac Arrest. Circulation. 2011;124(20):2225–32. 10.1161/CIRCULATIONAHA.110.015545. 22007075 10.1161/CIRCULATIONAHA.110.015545

[11] Kragh JFJ, Walters TJ, Baer DG, Fox CJ, Wade CE, Salinas J, et al. Survival With Emergency Tourniquet Use to Stop Bleeding in Major Limb Trauma. Ann Surg. 2009;249(1):1. 10.1097/SLA.0b013e31818842ba. 19106667 10.1097/SLA.0b013e31818842ba

[12] Ashour A, Cameron P, Bernard S, Fitzgerald M, Smith K, Walker T. Could bystander first-aid prevent trauma deaths at the scene of injury? Emerg Med Australasia. 2007;19(2):163–8. 10.1111/j.1742-6723.2007.00948.x. 10.1111/j.1742-6723.2007.00948.x 17448104

[13] Vaillancourt C, Grimshaw J, Brehaut JC, Osmond M, Charette ML, Wells GA, et al. A survey of attitudes and factors associated with successful cardiopulmonary resuscitation (CPR) knowledge transfer in an older population most likely to witness cardiac arrest: design and methodology. BMC Emerg Med. 2008;8(13):1–10. 10.1186/1471-227X-8-13. 18986547 10.1186/1471-227X-8-13 PMC2585573

[14] Brooks B, Chan S, Lander P, Adamson R, Hodgetts GA, Deakin CD. Public knowledge and confidence in the use of public access defibrillation. Heart. 2015;101(12):967. 10.1136/heartjnl-2015-307624. 25926599 10.1136/heartjnl-2015-307624

[15] Sasson C, Haukoos JS, Bond C, Rabe M, Colbert SH, King R, et al. Barriers and Facilitators to Learning and Performing Cardiopulmonary Resuscitation in Neighborhoods With Low Bystander Cardiopulmonary Resuscitation Prevalence and High Rates of Cardiac Arrest in Columbus. Circ Cardiovasc Qual Outcome. 2013;6(5):550–8. 10.1161/CIRCOUTCOMES.111.000097. 10.1161/CIRCOUTCOMES.111.000097 PMC3886185 24021699

[16] Nichols R, Horstman J. Recommendations for Improving Stop the Bleed: A Systematic Review. Mil Med. 2022;187(11–12):e1338–45. 10.1093/milmed/usac019. 35084491 10.1093/milmed/usac019

[17] Goolsby C, Jacobs L, Hunt RC, Goralnick E, Singletary EM, Levy MJ, et al. Stop the Bleed Education Consortium: Education program content and delivery recommendations. J Trauma Acute Care Surg. 2018;84(1):205. 10.1097/TA.0000000000001732. 29077676 10.1097/TA.0000000000001732

[18] Zideman DA, Singletary EM, Borra V, Cassan P, Cimpoesu CD, De Buck E, et al. European Resuscitation Council Guidelines 2021: First aid. Resuscitation. 2021;161:270–90. 10.1016/j.resuscitation.2021.02.013. 33773828 10.1016/j.resuscitation.2021.02.013

[19] European Commission. Next steps towards ‘Vision Zero’ – EU road safety policy framework 2021–2030. Publications Office; 2020. 10.2832/391271.

[20] De Buck E, Laermans J, Vanhove AC, Dockx K, Vandekerckhove P, Geduld H. An educational pathway and teaching materials for first aid training of children in sub-Saharan Africa based on the best available evidence. BMC Public Health. 2020;20(1). 10.1186/s12889-020-08857-5. Publisher: BioMed Central Ltd. Type: Article. 10.1186/s12889-020-08857-5 PMC7268765 32493323

[21] Hávold JI, Nesset E. From safety culture to safety orientation: Validation and simplification of a safety orientation scale using a sample of seafarers working for Norwegian ship owners. Saf Sci. 2009;47(3):305–26. 10.1016/j.ssci.2008.05.002.

[22] Riggs M, Franklin R, Saylany L. Associations between cardiopulmonary resuscitation (CPR) knowledge, self-efficacy, training history and willingness to perform CPR and CPR psychomotor skills: A systematic review. Resuscitation. 2019;138:259–72. 10.1016/j.resuscitation.2019.03.019. 30928504 10.1016/j.resuscitation.2019.03.019

[23] Sihvo M, Leena H, Tommi K. How to evaluate first aid skills after training: A systematic review. Scand J Trauma Resuscitation Emerg Med. 2022;30(1):56. 10.1186/s13049-022-01043-z. 10.1186/s13049-022-01043-z PMC9641962 36348427

[24] Farquharson B, Dixon D, Williams B, Torrens C, Philpott M, Laidlaw H, et al. The psychological and behavioural factors associated with laypeople initiating CPR for out-of-hospital cardiac arrest: A systematic review. BMC Cardiovasc Disord. 2023;23(1):19. 10.1186/s12872-022-02904-2. 36639764 10.1186/s12872-022-02904-2 PMC9840280

[25] Fratta KA, Bouland AJ, Vesselinov R, Levy MJ, Seaman KG, Lawner BJ, et al. Evaluating barriers to community CPR education. Am J Emerg Med. 2020;38(3):603–9. 10.1016/j.ajem.2019.10.019. 31866250 10.1016/j.ajem.2019.10.019

[26] Ndile ML, Saveman BI, Outwater AH, Mkoka DA, Backteman-Erlanson S. Implementing a layperson post-crash first aid training programme in Tanzania: A qualitative study of stakeholder perspectives. BMC Public Health. 2020;20(1). 10.1186/s12889-020-08692-8. 10.1186/s12889-020-08692-8 PMC7245810 32448350

[27] Ross EM, Redman TT, Mapp JG, Brown DJ, Tanaka K, Cooley CW, et al. Stop the Bleed: The Effect of Hemorrhage Control Education on Laypersons’ Willingness to Respond During a Traumatic Medical Emergency. Prehospital Disaster Med. 2018;33(2):127–32. 10.1017/S1049023X18000055. 29455698 10.1017/S1049023X18000055

[28] Pei L, Liang F, Sun S, Wang H, Dou H. Nursing students’ knowledge, willingness, and attitudes toward the first aid behavior as bystanders in traffic accident trauma: A cross-sectional survey. Int J Nurs Sci. 2019;6(1):65–9. 10.1016/j.ijnss.2018.11.003. 31406871 10.1016/j.ijnss.2018.11.003 PMC6608657

[29] Huy LD, Tung PT, Nhu LNQ, Linh NT, Tra DT, Thao NVP, et al. The willingness to perform first aid among high school students and associated factors in Hue. Vietnam PLoS ONE. 2022;17(7):e0271567. 10.1371/journal.pone.0271567. 35895665 10.1371/journal.pone.0271567 PMC9328566

[30] Abelsson A, Odestrand P, Nygårdh A. To strengthen self-confidence as a step in improving prehospital youth laymen basic life support. BMC Emerg Med. 2020;20(1):8. 10.1186/s12873-020-0304-8. 32000691 10.1186/s12873-020-0304-8 PMC6993316

[31] Hernández-Padilla J, Suthers F, Fernández-Sola C, Granero-Molina J. Development and psychometric assessment of the Basic Resuscitation Skills Self-Efficacy Scale. Eur J Cardiovasc Nurs. 2016;15(3):e10–8. 10.1177/1474515114562130. 25422522 10.1177/1474515114562130

[32] Roh YS, Issenberg SB, Chung HS, Kim SS. Development and psychometric evaluation of the Resuscitation Self-efficacy Scale for nurses. J Korean Acad Nurs. 2012;42(7):1079–86. 10.4040/jkan.2012.42.7.1079. 23377604 10.4040/jkan.2012.42.7.1079

[33] Morgado FF, Meireles JF, Neves CM, Amaral A, Ferreira ME. Scale development: Ten main limitations and recommendations to improve future research practices. Psicol Reflexão Crit. 2017;30. 10.1186/s41155-016-0057-1. 10.1186/s41155-016-0057-1 PMC6966966 32025957

[34] Vellante M, Baron-Cohen S, Melis M, Marrone M, Petretto DR, Masala C, et al. The “Reading the Mind in the Eyes” test: Systematic review of psychometric properties and a validation study in Italy. Cogn Neuropsychiatry. 2013;18(4):326–354. Publisher: Routledge. 10.1080/13546805.2012.721728. 10.1080/13546805.2012.721728 PMC6345369 23106125

[35] Chen G, Gully SM, Eden D. Validation of a New General Self-Efficacy Scale. Organ Res Methods. 2001;4(1):62–83. 10.1177/109442810141004.

[36] Bandura A. Self-efficacy: Toward a unifying theory of behavioral change. Adv Behav Res Ther. 1978;1(4):139–61. 10.1016/0146-6402(78)90002-4. 10.1037//0033-295x.84.2.191 847061

[37] Bandura A. Health promotion from the perspective of social cognitive theory. Psychol Health. 1998;13(4):623–49. 10.1080/08870449808407422.

[38] Gallagher MW. Self-Efficacy. In: Ramachandran VS, editor. Encyclopedia of Human Behavior (Second Edition). San Diego: Academic Press; 2012. pp. 314–320. 10.1016/B978-0-12-375000-6.00312-8.

[39] Bandura A. Social Foundations of Thought and Action: A Social Cognitive Theory. Prentice-Hall; 1986.

[40] Grether T, Sowislo JF, Wiese BS. Top-down or bottom-up? Prospective relations between general and domain-specific self-efficacy beliefs during a work-family transition. Pers Individ Differ. 2018;121:131–9. 10.1016/j.paid.2017.09.021.

[41] Arenas A, Cuadrado E, Castillo-Mayén R, Luque B, Rubio S, Gutiérrez-Domingo T, et al. Spanish validation of the cardiac self-efficacy scale: A gender invariant measure. Psychol Health Med. 2023;29(2):334–49. 10.1080/13548506.2023.2177683. 36782395 10.1080/13548506.2023.2177683

[42] Jonson CO, Pettersson J, Rybing J, Nilsson H, Prytz E. Short simulation exercises to improve emergency department nurses’ self-efficacy for initial disaster management: Controlled before and after study. Nurse Educ Today. 2017;55. 10.1016/j.nedt.2017.04.020. 10.1016/j.nedt.2017.04.020 28505521

[43] Bodys-Cupak I, Majda A, Zalewska-Puchała J, Kamińska A. The impact of a sense of self-efficacy on the level of stress and the ways of coping with difficult situations in Polish nursing students. Nurse Educ Today. 2016;45:102–7. 10.1016/j.nedt.2016.07.004. 27429414 10.1016/j.nedt.2016.07.004

[44] Garaika Margahana H, Efficacy Self. Self Personality and Self Confidence on Entrepreneurial Intention: Study on Young Enterprises. J Entrep Educ. 2019;22(1):1–12.

[45] González-Salvado V, Abelairas-Gómez C, Peña-Gil C, Neiro-Rey C, Barcala-Furelos R, González-Juanatey JR, et al. Basic life support training into cardiac rehabilitation programs: A chance to give back. A community intervention controlled manikin study. Resuscitation. 2018;127:14–20. 10.1016/j.resuscitation.2018.03.018. 10.1016/j.resuscitation.2018.03.018 29545137

[46] Nykänen M, Salmela-Aro K, Tolvanen A, Vuori J. Safety self-efficacy and internal locus of control as mediators of safety motivation – Randomized controlled trial (RCT) study. Saf Sci. 2019;117:330–8. 10.1016/j.ssci.2019.04.037.

[47] Moshki M, Ghofranipour F, Hajizadeh E, Azadfallah P. Validity and reliability of the multidimensional health locus of control scale for college students. BMC Public Health. 2007;7. 10.1186/1471-2458-7-295. 10.1186/1471-2458-7-295 PMC2206030 17942001

[48] Ali MIEB, Habib NS, Sharaa HM. Effect of First Aid Training Program on Construction Workers’ Self-Efficacy in Egypt. P J M H S. 2021;15(1):403–6.

[49] Ho JKM, Chung JYS, Cheung SN, Pang WWY, Yau PY, Lam SC. Self-efficacy of emergency management of domestic helpers in pediatric home accidents: A cross-sectional survey in Hong Kong. Front Pediatr. 2022;10:997834. 36340717 10.3389/fped.2022.997834 PMC9627280

[50] Tatebe L, Speedy S, Kang D, Barnum T, Cosey-Gay F, Regan S, et al. Empowering Bystanders to Intervene: Trauma Responders Unify to Empower (TRUE) Communities. J Surg Res. 2019;238:255–64. 10.1016/j.jss.2019.02.029. 30954087 10.1016/j.jss.2019.02.029

[51] Schunk DH, DiBenedetto MK. Self-efficacy and human motivation. Adv Motiv Sci. 2021;8:153–79.

[52] Page MJ, McKenzie JE, Bossuyt PM, Boutron I, Hoffmann TC, Mulrow CD, et al. The PRISMA 2020 statement: An updated guideline for reporting systematic reviews. BMJ. 2021;372:n71. 10.1136/bmj.n71. 33782057 10.1136/bmj.n71 PMC8005924

[53] Wei YL, Chen LL, Li TC, Ma WF, Peng NH, Huang LC. Self-efficacy of first aid for home accidents among parents with 0- to 4-year-old children at a metropolitan community health center in Taiwan. Accid Anal Prev. 2013;52:182–7. 10.1016/j.aap.2012.12.002. 23348100 10.1016/j.aap.2012.12.002

[54] Polloni L, Baldi I, Lazzarotto F, Bonaguro R, Toniolo A, Celegato N, et al. School personnel’s self-efficacy in managing food allergy and anaphylaxis. Pediatr Allergy Immunol Off Publ Eur Soc Pediatr Allergy Immunol. 2016;27(4):356–60. 10.1111/pai.12550. 10.1111/pai.12550 26887784

[55] Wallace JM, Harris KM, Stankovich J, Ayton J, Bettiol SS. Emergency first aid readiness in Antarctica: Australian Antarctic expeditioners’ first aid credentials and self-efficacy. Emerg Med Australasia. 2020;32(1):67–74. 10.1111/1742-6723.13339. 10.1111/1742-6723.13339 31268242

[56] D’Angelo JJ, Ritchie SD, Little JR, Johnson DE, Vanderburgh D, Orkin AM, et al. Validating the Remote First Aid Self-Efficacy Scale for Use in Evaluation and Training of First Responders in Remote Contexts. Wilderness Environ Med. 2023;34(1):15–21. 10.1016/j.wem.2022.09.006. 36446725 10.1016/j.wem.2022.09.006

[57] Alcázar Artero PM, Pardo Rios M, Greif R, Ocampo Cervantes AB, Gijón-Nogueron G, Barcala-Furelos R, et al. Efficiency of virtual reality for cardiopulmonary resuscitation training of adult laypersons: A systematic review. Medicine. 2023;102(4). 10.1097/MD.0000000000032736. 10.1097/MD.0000000000032736 PMC9875948 36705392

[58] Liu Q, Tang Q, Wang Y. The effects of pretraining intervention in immersive embodied virtual reality cardiopulmonary resuscitation training. Behav Inform Technol. 2021;40(12):1265–77. 10.1080/0144929X.2021.1960606.

[59] Ge P, Zhang J, Lyu K, Niu Y, Li Q, Xiong P, et al. The current status and factors related to the preparation of home first-aid kits in China. Front Public Health. 2022;10:1036299. 36518576 10.3389/fpubh.2022.1036299 PMC9742271

[60] Luszczynska A, Gutiérrez-Doña B, Schwarzer R. General self-efficacy in various domains of human functioning: Evidence from five countries. Int J Psychol. 2005;40(2):80–9.

[61] Buttussi F, Chittaro L, Valent F. A virtual reality methodology for cardiopulmonary resuscitation training with and without a physical mannequin. J Biomed Inform. 2020;111:103590. 10.1016/j.jbi.2020.103590. 33039589 10.1016/j.jbi.2020.103590

[62] Seifi OSE, Mortada EM, Abdo NM. Effect of community-based intervention on knowledge, attitude, and self-efficacy toward home injuries among Egyptian rural mothers having preschool children. PLoS ONE. 2018;13(6):e0198964. 10.1371/journal.pone.0198964. 29927950 10.1371/journal.pone.0198964 PMC6013117

[63] Heggestad ED, Scheaf DJ, Banks GC, Monroe Hausfeld M, Tonidandel S, Williams EB. Scale Adaptation in Organizational Science Research: A Review and Best-Practice Recommendations. J Manag. 2019;45(6):2596–627. 10.1177/0149206319850280.

[64] Wulff JN, Sajons GB, Pogrebna G, Lonati S, Bastardoz N, Banks GC, et al. Common methodological mistakes Leadersh Q. 2023;34(1):101677. 10.1016/j.leaqua.2023.101677.

[65] Polloni L, Baldi I, Lazzarotto F, Bonaguro R, Toniolo A, Gregori D, et al. Multidisciplinary education improves school personnel’s self- efficacy in managing food allergy and anaphylaxis. Pediatr Allergy Immunol. 2020;31(4):380–7. 10.1111/pai.13212. 31943386 10.1111/pai.13212

[66] Yu YC, Liang JC. Relationships among Affect, Hardiness and Self-Efficacy in First Aid Provision by Airline Cabin Crew. Int J Environ Res Public Health. 2021;18(4):2108. 10.3390/ijerph18042108. 33671508 10.3390/ijerph18042108 PMC7926649

[67] Liu ZM, Fan X, Liu Y, Ye Xd. Effects of immersive virtual reality cardiopulmonary resuscitation training on prospective kindergarten teachers’ learning achievements, attitudes and self-efficacy. Br J Educ Technol. 2022;53(6):2050–2070. 10.1111/bjet.13237.

[68] Schumann SA, Schimelpfenig T, Sibthorp J, Collins RH. An Examination of Wilderness First Aid Knowledge, Self-Efficacy, and Skill Retention. Wilderness Environ Med. 2012;23(3):281–7. 10.1016/j.wem.2012.04.005. 22857870 10.1016/j.wem.2012.04.005

[69] Shafer PO, Gilchrist B, Miller W, Owens S, Ficker D, Haynes-Smith L, et al. Improving self-efficacy in seizure first aid: Developing a seizure first aid certification program in the United States. Epilepsy Behav. 2022;129:108624. 10.1016/j.yebeh.2022.108624. 35247833 10.1016/j.yebeh.2022.108624

[70] Yoon W, Ro YS, Cho Si. A mediation analysis of the effect of practical training on the relationship between demographic factors, and bystanders’ self-efficacy in CPR performance. PLoS ONE. 2019;14(4):e0215432. 10.1371/journal.pone.0215432. 10.1371/journal.pone.0215432 PMC6488056 31034486

[71] Muise J, Oliver E, Newell P, Forsyth M. Improving individuals’ propensity to act in a medical emergency: A quasi-randomised trial to test the impact of a learning intervention. Health Educ J. 2019;78(2):214–25. 10.1177/0017896918796030.

[72] Avau B, Vanhove AC, Scheers H, Stroobants S, Lauwers K, Vandekerckhove P, et al. Impact of the Use of Simulated Patients in Basic First Aid Training on Laypeople Knowledge, Skills, and Self-efficacy: A Controlled Experimental Study. Simul Healthc. 2022;17(4):213–9. 10.1097/SIH.0000000000000657. 35921627 10.1097/SIH.0000000000000657 PMC9351698

[73] Ro YS, Shin SD, Song KJ, Hong SO, Kim YT, Cho SI. Bystander cardiopulmonary resuscitation training experience and self-efficacy of age and gender group: A nationwide community survey. Am J Emerg Med. 2016;34(8):1331–7. 10.1016/j.ajem.2015.12.001. 27037129 10.1016/j.ajem.2015.12.001

[74] Ro YS, Do Shin S, Song KJ, Hong SO, Kim YT, Lee DW, et al. Public awareness and self-efficacy of cardiopulmonary resuscitation in communities and outcomes of out-of-hospital cardiac arrest: A multi-level analysis. Resuscitation. 2016;102:17–24. 26898411 10.1016/j.resuscitation.2016.02.004

[75] Buckler DG, Almodovar A, Snobelen P, Abella BS, Blewer A, Leary M. Observing the stages of bystander intervention in virtual reality simulation. World J Emerg Med. 2019;10(3):145–51. 10.5847/wjem.j.1920-8642.2019.03.003. 31171944 10.5847/wjem.j.1920-8642.2019.03.003 PMC6545373

[76] Neff A, Niessen C, Sonnentag S, Unger D. Expanding crossover research: The crossover of job-related self-efficacy within couples. Hum Relat Stud Towards Integr Soc Sci. 2013;66(6):803–27. 10.1177/0018726712465095.

[77] Lind N, Hansson H, Lagerkvist CJ. Development and validation of a measurement scale for self-efficacy for farmers’ mastitis prevention in dairy cows. Prev Vet Med. 2019;167:53–60. 10.1016/j.prevetmed.2019.03.025. 31027722 10.1016/j.prevetmed.2019.03.025

[78] Foulstone AR, Kelly A. Enhancing academic self-efficacy and performance among fourth year psychology students: Findings from a short educational intervention. Int J Sch Teach Learn. 2019;13(2):1–9. 10.20429/ijsotl.2019.130209.

[79] Choo CC, Devakaran B, Chew PKH, Zhang MWB. Smartphone Application in Postgraduate Clinical Psychology Training: Trainees’ Perspectives. Int J Environ Res Public Health. 2019;16(21):4206. 10.3390/ijerph16214206. 31671592 10.3390/ijerph16214206 PMC6862580

[80] Pachler D, Kuonath A, Frey D. How transformational lecturers promote students’ engagement, creativity, and task performance: The mediating role of trust in lecturer and self-efficacy. Learn Individ Differ. 2019;69:162–72. 10.1016/j.lindif.2018.12.004.

[81] Lee C, Payne LL, Berdychevsky L. The Roles of Leisure Attitudes and Self-Efficacy on Attitudes Toward Retirement Among Retirees: A Sense of Coherence Theory Approach. Leis Sci. 2020;42(2):152–69. 10.1080/01490400.2018.1448025.

[82] Bouton E, Tal SB, Asterhan CSC. Students, social network technology and learning in higher education: Visions of collaborative knowledge construction vs. the reality of knowledge sharing. Internet High Educ. 2021;49:100787. 10.1016/j.iheduc.2020.100787.

[83] Di W, Nie Y, Chua BL, Chye S, Teo T. Developing a Single-Item General Self-Efficacy Scale: An Initial Study. J Psychoeduc Assess. 2023;41(5):583–98. 10.1177/07342829231161884.

[84] Chen G, Gully SM, Eden D. General self-efficacy and self-esteem: Toward theoretical and empirical distinction between correlated self-evaluations. J Organ Behav. 2004;25(3):375–95. 10.1002/job.251.

[85] Scherbaum CA, Cohen-Charash Y, Kern MJ. Measuring General Self-Efficacy: A Comparison of Three Measures Using Item Response Theory. Educ Psychol Meas. 2006;66(6):1047–63. 10.1177/0013164406288171.

[86] Heilala V, Kelly R, Saarela M, Jääskelä P, Kärkkäinen T. The Finnish Version of the Affinity for Technology Interaction (ATI) Scale: Psychometric Properties and an Examination of Gender Differences. Int J Hum Comput Interac. 2023;39(4):874–92. 10.1080/10447318.2022.2049142.

[87] Lubbe D. Parallel analysis with categorical variables: Impact of category probability proportions on dimensionality assessment accuracy. Psychol Methods. 2019;24(3):339–51. 10.1037/met0000171. 29745684 10.1037/met0000171

[88] Brown TA. In: Confirmatory Factor Analysis for Applied Research. 2nd ed. Methodology in the Social Sciences. New York: Guilford Publications; 2015.

[89] Sijtsma K, Pfadt JM. Part II: On the Use, the Misuse, and the Very Limited Usefulness of Cronbach’s Alpha: Discussing Lower Bounds and Correlated Errors. Psychometrika. 2021;86(4):843–60. 10.1007/s11336-021-09789-8. 34387809 10.1007/s11336-021-09789-8 PMC8636457

[90] Sijtsma K, van der Ark LA. A tutorial on how to do a Mokken scale analysis on your test and questionnaire data. Br J Math Stat Psychol. 2017;70(1):137–58. 10.1111/bmsp.12078. 27958642 10.1111/bmsp.12078

[91] Sijtsma K, Meijer RR, Andries van der Ark L. Mokken scale analysis as time goes by: An update for scaling practitioners. Personality Individ Differ. 2011;50(1):31–37. 10.1016/j.paid.2010.08.016.

[92] Mokken RJ. A Theory and Procedure of Scale Analysis: With Applications in Political Research. Berlin: Walter de Gruyter; 1971. 10.1515/9783110813203.

[93] Wu H, Estabrook R. Identification of Confirmatory Factor Analysis Models of Different Levels of Invariance for Ordered Categorical Outcomes. Psychometrika. 2016;81(4):1014–45. 10.1007/s11336-016-9506-0. 27402166 10.1007/s11336-016-9506-0 PMC5458787

[94] Svetina D, Rutkowski L, Rutkowski D. Multiple-Group Invariance with Categorical Outcomes Using Updated Guidelines: An Illustration Using Mplus and the lavaan/semTools Packages. Struct Equ Model Multidiscip J. 2020;27(1):111–30. 10.1080/10705511.2019.1602776.

[95] Auerswald M, Moshagen M. How to determine the number of factors to retain in exploratory factor analysis: A comparison of extraction methods under realistic conditions. Psychol Methods. 2019;24(4):468–91. 10.1037/met0000200. 30667242 10.1037/met0000200

[96] Buja A, Eyuboglu N. Remarks on Parallel Analysis. Multivar Behav Res. 1992;27(4):509–40. 10.1207/s15327906mbr2704_2. 10.1207/s15327906mbr2704_2 26811132

[97] Garrido LE, Abad FJ, Ponsoda V. Performance of Velicer’s Minimum Average Partial Factor Retention Method With Categorical Variables. Educ Psychol Meas. 2011;71(3):551–70. 10.1177/0013164410389489.

[98] Holgado-Tello FP, Chacón-Moscoso S, Barbero-García I, Vila-Abad E. Polychoric versus Pearson correlations in exploratory and confirmatory factor analysis of ordinal variables. Qual Quant. 2010;44(1):153–66. 10.1007/s11135-008-9190-y.

[99] El-Sheikh AA, Abonazel MR, Gamil N. A review of software packages for structural equation modeling: A comparative study. Appl Math Phys. 2017;5(3):85–94. 10.12691/amp-5-3-2.

[100] Beauducel A, Herzberg PY. On the Performance of Maximum Likelihood Versus Means and Variance Adjusted Weighted Least Squares Estimation in CFA. Struct Equ Model Multidiscip J. 2006;13(2):186–203. 10.1207/s15328007sem1302_2.

[101] DiStefano C, Morgan GB. A Comparison of Diagonal Weighted Least Squares Robust Estimation Techniques for Ordinal Data. Struct Equ Model Multidiscip J. 2014;21(3):425–38. 10.1080/10705511.2014.915373.

[102] Foldnes N, Grønneberg S. The sensitivity of structural equation modeling with ordinal data to underlying non-normality and observed distributional forms. Psychol Methods. 2021. 10.1037/met0000385. 33793270 10.1037/met0000385

[103] Forero CG, Maydeu-Olivares A, Gallardo-Pujol D. Factor Analysis with Ordinal Indicators: A Monte Carlo Study Comparing DWLS and ULS Estimation. Struct Equ Model Multidiscip J. 2009;16(4):625–41. 10.1080/10705510903203573.

[104] MacCallum RC, Widaman KF, Preacher KJ, Hong S. Sample Size in Factor Analysis: The Role of Model Error. Multivar Behav Res. 2001;36(4):611–37. 10.1207/S15327906MBR3604_06. 10.1207/S15327906MBR3604_06 26822184

[105] Hu L, Bentler PM. Cutoff criteria for fit indexes in covariance structure analysis: Conventional criteria versus new alternatives. Struct Equ Model Multidiscip J. 1999;6(1):1–55. 10.1080/10705519909540118.

[106] Loevinger J. The technic of homogeneous tests compared with some aspects of scale analysis and factor analysis. Psychol Bull. 1948;45(6):507–29. 10.1037/h0055827. 18893224 10.1037/h0055827

[107] Sijtsma K, Hemker BT. A Taxonomy of IRT Models for Ordering Persons and Items Using Simple Sum Scores. J Educ Behav Stat. 2000;25(4):391–415. 10.3102/10769986025004391.

[108] Koo TK, Li MY. A Guideline of Selecting and Reporting Intraclass Correlation Coefficients for Reliability Research. J Chiropr Med. 2016;15(2):155–63. 10.1016/j.jcm.2016.02.012. 27330520 10.1016/j.jcm.2016.02.012 PMC4913118

[109] Nunnally JC, Bernstein IH. Psychometric theory 3E. 3rd ed. New York: McGraw-Hill; 1994.

[110] Gosling SD, Rentfrow PJ, Swann WB. A very brief measure of the Big-Five personality domains. J Res Pers. 2003;37(6):504–28. 10.1016/S0092-6566(03)00046-1.

[111] Lönnqvist JE, Verkasalo M, Leikas S. Viiden suuren persoonallisuusfaktorin 10, 60, ja 300 osion julkiset mittarit. Psykologia. 2008;43(5):328–41.

[112] van der Ark LA. Mokken scale analysis in R. J Stat Softw. 2007;20(11):1–19. 10.18637/jss.v020.i11.

[113] Straat JH, van der Ark LA, Sijtsma K. Using Conditional Association to Identify Locally Independent Item Sets. Methodol Eur J Res Methods Behav Soc Sci. 2016;12(4):117–23. 10.1027/1614-2241/a000115.

[114] Ligtvoet R, Van der Ark LA, Te Marvelde JM, Sijtsma K. Investigating an invariant item ordering for polytomously scored items. Educ Psychol Meas. 2010;70(4):578–95.

[115] Satorra A, Bentler PM. A scaled difference chi-square test statistic for moment structure analysis. Psychometrika. 2001;66(4):507–14. 10.1007/BF02296192. 10.1007/s11336-009-9135-y PMC2905175 20640194

[116] Ponterotto JG, Fietzer AW, Fingerhut EC, Woerner S, Stack L, Magaldi-Dopman D, et al. Development and Initial Validation of the Multicultural Personality Inventory (MPI). J Pers Assess. 2014;96(5):544–58. 10.1080/00223891.2013.843181. 24206108 10.1080/00223891.2013.843181

[117] Lazić M, Jovanović V, Gavrilov-Jerković V. The general self-efficacy scale: New evidence of structural validity, measurement invariance, and predictive properties in relationship to subjective well-being in Serbian samples. Curr Psychol. 2021;40(2):699–710. 10.1007/s12144-018-9992-6.

[118] Pulkka AT, Budlong L. Associations Between Achievement Goal Orientations, Preferred Learning Practices, and Motivational Evaluations of Learning Environment Among Finnish Military Reservists. Front Psychol. 2022;13:902478. 10.3389/fpsyg.2022.902478. 35814131 10.3389/fpsyg.2022.902478 PMC9262099

[119] Carlson KD, Herdman AO. Understanding the Impact of Convergent Validity on Research Results. Organ Res Methods. 2012;15(1):17–32. 10.1177/1094428110392383.

[120] Bosco FA, Aguinis H, Singh K, Field JG, Pierce CA. Correlational effect size benchmarks. J Appl Psychol. 2015;100(2):431–49. 10.1037/a0038047. 25314367 10.1037/a0038047

[121] Barańczuk U. The Five-Factor Model of Personality and Generalized Self Efficacy. J Individ Differ. 2021;42(4):183–93. 10.1027/1614-0001/a000345.

[122] Boateng GO, Neilands TB, Frongillo EA, Melgar-Quiñonez HR, Young SL. Best Practices for Developing and Validating Scales for Health, Social, and Behavioral Research: A Primer. Front Public Health. 2018;6(149):1–18. 10.3389/fpubh.2018.00149. 29942800 10.3389/fpubh.2018.00149 PMC6004510

[123] World Health Organization. Save LIVES - A road safety technical package. 2017. https://iris.who.int/bitstream/handle/10665/255199/9789241511704-eng.pdf?sequence=1. Accessed 20 Mar 2025.

[124] Jacobs L, Keating JJ, Hunt RC, Butler FK, Pons PT, Gestring M, et al. Stop the Bleed^®^. Curr Probl Surg. 2022;59(10):101193. 10.1016/j.cpsurg.2022.101193. 36253022 10.1016/j.cpsurg.2022.101193

[125] Oliver E, Cooper J, McKinney D. Can first aid training encourage individuals’ propensity to act in an emergency situation? A pilot study Emerg Med J. 2014;31(6):518–20. 10.1136/emermed-2012-202191. 23811862 10.1136/emermed-2012-202191

[126] Van de Velde S, Roex A, Vangronsveld K, Niezink L, Van Praet K, Heselmans A, et al. Can training improve laypersons helping behaviour in first aid? A randomised controlled deception trial. Emerg Med J. 2013;30(4):292–7. 10.1136/emermed-2012-201128. 22562070 10.1136/emermed-2012-201128

[127] Pellegrino JL, Smith SE, Banton E, Sudhir A. Systematic Review of Lay Responders Educational Outcomes to Identify Life-Threatening Bleeding. Int J First Aid Educ. 2022;4(2). 10.25894/ijfae.4.2.4.

[128] Oliver E, Forsyth M, Colebourn D, Gordon E, Taylor H, Mulligan J. A randomized trial of blended first aid education for the public. Int J First Aid Educ. 2020;3(1). Publisher: Aperio Press. 10.25894/ijfae.3.1.7.

[129] Pellegrino JL, Charlton N, Goolsby C. “Stop the Bleed” Education Assessment Tool (SBEAT): Development and Validation. Cureus. 2020;12(9):e10567. 10.7759/cureus.10567. 10.7759/cureus.10567 PMC7577301 33101813

[130] Podsakoff PM, MacKenzie SB, Lee JY, Podsakoff NP. Common method biases in behavioral research: A critical review of the literature and recommended remedies. J Appl Psychol. 2003;88(5):879. 14516251 10.1037/0021-9010.88.5.879

