# Supplementary material for: First aid self-efficacy: a scale adaptation and psychometric properties
Source: BMC Public Health. 2025 Apr 1;25:1234. doi: 10.1186/s12889-025-22486-w (PMC11963637; doi:10.1186/s12889-025-22486-w)
Supplement: Supplementary file 1 — Questionnaire instruments. [file 12889_2025_22486_MOESM1_ESM.pdf]

Finnish version of the First Aid Self-Efficacy Scale

[FI] Starts

Seuraavaksi sinulta kysytään kahdeksan ensiapuun liittyvää kysymystä.

Arvioi seuraavien väittämien avulla toimintaasi erilaisissa ensiapua vaativissa tilanteissa.
1 = Vahvasti eri mieltä; 2 = Eri mieltä; 3 = Ei samaa eikä eri mieltä ; 4 = Samaa mieltä;
5 = Vahvasti samaa mieltä

Kysymykset I

1. Pystyisin auttamaan useimmissa ensiaputilanteissa.
2. Olen varma, että suoriutuisin haastavista ensiaputilanteista alusta loppuun asti.
3. Yleisesti ottaen pystyn mielestäni saamaan aikaan tuloksia ensiaputilanteissa.
4. Uskon onnistuvani missä tahansa ensiaputilanteessa.
5. Pystyisin auttamaan onnistuneesti monissa ensiaputilanteissa.
6. Luotan siihen, että pystyn toimimaan epäröimättä monissa ensiaputilanteissa.
7. Verrattuna muihin ihmisiin hallitsen erittäin hyvin useimmat ensiaputilanteet.
8. Pystyn suoriutumaan melko hyvin tiukoissakin ensiaputilanteissa.

[FI] ends

Study II Questionnaires II

Part 1: Self-efficacy (same as Study 1)

Part 2: Knowledge of first aid

The questionnaire was provided in Finnish to the participants. The English questionnaire is a translated version of the original Finnish version.

FI: Seuraavaksi sinulta kysytään kuusi ensiaputaitoihin liittyvää kysymystä. On tärkeää, että vastaat mahdollisimman rehellisesti käyttämällä vain omaa osaamistasi ilman ulkoisia lähteitä.
Huomaathan, että osassa vastauksista voi olla myös useampi kuin yksi oikea vaihtoehto!
EN: Next, you will be asked six questions related to first aid skills. It is important that you answer as honestly as possible, using only your own knowledge and no external sources.
Please note that some of the answers may also have more than one correct option!

1. FI: Saavut liikenneonnettomuustilanteeseen, jossa auto on ajanut ulos tieltä. Hätänumeroon on jo soitettu, mutta et saa autossa istuvaa kuljettajaa hereille. Mitä teet?
   EN: *You arrive at the scene of a traffic accident where the car has driven off the road. The emergency services have already been called, but you cannot get the driver to wake up. What would you do?*

- [ ] Tarkistan autettavan hengityksen ja jään pitämään hengitysteitä auki ammattiavun saapumiseen asti (*I check if the driver is breathing and stay to keep his or her airways open until the professionals arrive).*

- Siirrän autettavan autosta, jotta saan tarkistettua hengityksen ja käännettyä hänet kylkiasentoon *(I move the driver out of the car, so that I can check the drivers´ breathing and I can move her/him in the recovery position*).
- Tarkistan autettavan hengityksen ja lähden hakemaan lisää apua, jotta saan siirrettyä autettavan pois autosta mahdollisimman *nopeasti (check the drivers´ breathing and go to get more help, so that I can move the driver out of the car as soon as possible).*
- Odotan avun tuloa paikalle ja huolehdin siitä, että kukaan ei koske autettavaan, jotta mahdolliset vammat eivät pahene *(I wait until help arrives and I make sure that nobody touches the driver to prevent more injuries).*
- En tiedä mitä tekisin, enkä halua arvailla. *(I do not know what I would do and I do not want to guess).*

2. FI: Läheisellesi tulee yllättäen huono olo. Et saa läheisesi puheesta selvää ja toinen käsi roikkuu voimattomana. Mitä huomioisit tilanteessa?
   EN: Suddenly, your close family member does not feel well. You cannot understand her or his speech and one of their hands is hanging weakly. What would you consider in the situation?

- Tilaan liittyy sana FAST *(The evaluation tool FAST is connected to this situation).*
- On ensiarvoisen tärkeää tunnistaa oireet. *(It is crucial to recognise symptoms).*
- On välittömästi lähdettävä viemään henkilö lääkäriin *(The person has to be taken to the doctor immediately).*
- Muita oireita voivat olla, suunpielen roikkuminen ja tasapainohäiriöt (*The other symptoms can be that the mouth has dropped on one side or they have problems with balance).*
- Syynä on useimmiten aivojen verisuonen tukos *(The reason for the symptoms is mostly a blocked blood vessel).*
- Tilanteessa tulee poikkeuksetta soittaa 112 (*In this situation the emergency services must be alerted immediately without exception).*
- En tiedä, mitä pitäisi huomioida enkä halua arvailla (*I don´t know what I would do and I don´t want to guess).*

3. FI: Diabetesta sairastava naapurisi nojaa huonovointisena postilaatikkoon. Hän on kalpea ja hieman äreän oloinen. Miten toimit?
   EN: Your neghbour who has diabetes is not feeling well and is leaning on the mail box. They are pale and a little bit grumpy. What would you do?

- Haen autettavalle lasin vettä ja ohjaan istumaan *(I would take a glass of water to the person and help the person to sit down).*
- Soitan välittömästi 112 ja ohjaan autettavan istumaan *(I will call the emergency services and help the person to sit down).*
- Ohjaan autettavan sisälle ja annan hänelle sokeripitoista juotavaa tai syötävää (I will help the person inside and give the person something sugary to drink or eat).
- Mikäli autettavan tila ei parane syömisen jälkeen, soitan 112 *(If the person does not feel well, I will call the emergency sevices).*
- Ohjaan autettavan sisälle ja autan häntä pistämään välittömästi insuliinia *(I help the person inside and help the person to inject insulin immediately).*
- En tiedä, mitä pitäisi huomioida enkä halua arvailla *(I don´t know what I would do and I don´t want to guess).*

4. FI: Defibrillaattoria käytettäessä olennaista on, että…
   EN: When the defibrillator is needed it is essential that...

- ☐ liimaelektrodit kiinnitetään rintakehällä oikean solisluun alle sekä vasemman kyljen kohdalle (The place of the pads are under the right clavicle and to the left side of the torso).
- ☐ liimaelektrodit kiinnitetään rintakehällä vasemman solisluun alle sekä oikean kyljen kohdalle (*The place of the pads are under the left clavicle and to the right side of the torso).*
- ☐ laitteen antaman sähköiskun aikana on hengenvaarallista olla kosketuksessa autettavaan *(When the defibrillator gives the electric shock it is life threatening to touch the person who needs help).*
- ☐ defibrilloinnin jälkeen painelu-puhalluselvytys aloitetaan tarvittaessa mahdollisimman nopeasti uudelleen *(After the defibrillation, start the cardiac pulmonary resusciation CPR if needed as soon as possible).*
- ☐ Defibrillaattorin käyttö ei estä nopeaa painelu-puhalluselvytyksen aloittamista *(The use of the defibrillator would not prevent the immediate start of the CPR).*
- ☐ laitetta voi käyttää kuka tahansa (*Anyone can use the defibrillator).*
- ☐ En tiedä, mitä pitäisi huomioida enkä halua arvailla *(I don´t know what I should consider and I don´t want to guess).*

5. FI: Elvytystilanteessa paineluiden ja puhalluksen suhde mielestäsi on…
   EN: In the situation of the resuscitation in my mind the ratio of pressures and blows are ...

- ☐ 15 painelua 2 puhallusta *(15 pressures 2 blows).*
- ☐ 15 painelua, ei puhalluksia *(15 pressures no blows).*
- ☐ 30 painelua 2 puhallusta *(30 pressures 2 blows).*
- ☐ 30 painelua 5 puhallusta *(30 pressures 5 blows).*
- ☐ en tiedä, enkä halua arvailla *(I don´t know what I would do and I don´t want to guess).*

6. FI: Tajuttoman autettavan suurin henkeä uhkaava vaara mielestäsi on...
   EN: In your opinion, the greatest life-threatening danger to an unconscious person is.
   .

- ☐ Tukehtuminen (Choking).
- ☐ Paleltuminen (Hyphotermia).
- ☐ Oksentaminen (Vomitting).
- ☐ Sydämen pysähtyminen (Cardiac arrest).
- ☐ Syvempään tajuttomuuteen vajoaminen (deeper unconsciousness).
- ☐ Halvaantuminen (Stroke).
- ☐ En tiedä, enkä halua arvailla *(I don´t know what I would do and I don´t want to guess*).

Part 3: The personality traits

1. Gosling, S. D., Rentfrow, P. J., & Swann, W. B., Jr. (2003). A Very Brief Measure of the Big Five Personality Domains. *Journal of Research in Personality, 37, 504-528.*
2. Lönnqvist, J-E., Verkasalo M. & Leikas, S. (2008). Viiden suuren persoonallisuusfaktorin 10, 60, ja 300 osion julkiset mittarit. Psykologia, 43, 328-341.

Part 4 (the level of self-efficacy)

New General Self-Efficacy Scale by Chen, Gully, and Eden (2001) translated into Finnish to the participants.

[FI] Starts

Missä määrin olet samaa tai eri mieltä seuraavien väitteiden kanssa?
1 = Vahvasti eri mieltä; 2 = Eri mieltä; 3 = Ei samaa eikä eri mieltä; 4 = Samaa mieltä;
5 = Vahvasti samaa mieltä.

1. Pystyn saavuttamaan suurimman osan itselleni asettamistani tavoitteista.
2. Kohdatessani hankalia tehtäviä olen varma, että saan ne suoritettua.
3. Olen yleisesti sitä mieltä, että pystyn saamaan aikaan minulle merkityksellisiä lopputuloksia.
4. Uskon onnistuvani lähes missä tahansa pyrkimyksessäni, johon päättäväisesti paneudun.
5. Pystyn onnistuneesti voittamaan monia haasteita.
6. Olen varma, että pystyn suoriutumaan tehokkaasti monissa erilaisissa tehtävissä.
7. Pystyn tekemään useimmat asiat todella hyvin verrattuna muihin ihmisiin.
8. Pystyn suoriutumaan varsin hyvin jopa tiukoissa tilanteissa.

[FI] Ends

Part 5

Questionnaire by Abelsson et. al. (2020) translated into Finnish to the participants.

[FI] Starts

Lopuksi sinulta kysytään seitsemän ensiaputilanteessa toimimiseen liittyvää kysymystä. Valitse liukukytkimellä lähinnä omaa tuntemustasi kuvaava arvo siten, että arvo 1 kuvaa vähiten omaa kokemustasi ja arvo 100 eniten.
Huomaathan liikuttaa kytkintä ennen valintaasi, jotta arvo tallentuu.

1. Tiedän, miten tunnistan henkilön, jolla on äkillinen sydänpysähdys.
2. Kykenisin antamaan paineluelvytystä sydänpysähdyksen saaneelle.
3. Kykenisin antamaan puhalluselvytystä sydänpysähdyksen saaneelle.
4. En pelkäisi aiheuttavani haittaa henkilölle, jolle antaisin painelu-puhalluselvytystä.
5. Käyttäisin defibrillaattoria eli sydäniskuria, jos laite olisi saatavilla elvytystilanteessa.
6. *Tietäisin millaista ensiapua antaisin henkilölle, joka olisi loukkaantunut onnettomuudessa.*
7. *Antaisin ensiapua, mikäli henkilö olisi loukkaantunut onnettomuudessa.*

[FI] Ends

Study 3 (English version of the first aid self-efficacy scale)

1 I would be able to help in most first aid situations.
2 I am sure that I would be able to handle challenging first aid situations from start to finish.
3 Generally speaking, I think that I can create results in first aid situations.
4 I believe I can succeed in any first aid situation.
5 I would be able to successfully provide help in many first aid situations.

6 I am confident that I can provide help without hesitation in many first aid situations.
7 In comparison to other people, I can handle most first aid situations very well.
8 I am able to perform quite well even in challenging first aid situation.
